# Supplementary material for: Risk of hand and forearm conditions due to vibrating hand-held tools exposure: a retrospective cohort study from Sweden
Source: BMJ Open. 2024 Jun 18;14(6):e080777. doi: 10.1136/bmjopen-2023-080777 (PMC11191761; doi:10.1136/bmjopen-2023-080777)
Supplement: Supplementary data [file bmjopen-2023-080777supp001.pdf]

Supplemental table 1. Baseline characteristics of all included individuals that reported exposure to vibrating hand-held tools as "not at all", "some" and "much".

	"Does your work involve working with vibrating hand-held tools?"			
	**"Not at all"**	**"Some"**	**"Much"**	**P-value**
	**12,220 (85%)**	**1,392 (10%)**	**730 (5%)**	
Age (years)	57±6	57±6	57±6	0.18
Sex (men)	4845 (40%)	1003 (72%)	601 (82%)	**<0.001**†,‡,§
Current smoking	3458 (28%)	477 (34%)	257 (35%)	**<0.001**†,‡
Antihypertensive treatment	5763 (47%)	708 (51%)	373 (51%)	**0.005**†,‡
Prevalent diabetes	546 (5%)	85 (6%)	37 (5%)	**0.02**†
Alcohol consumption (g/day)	7.3 [1.7-15.5]	8.1 [1.8-17.3]	9.0 [1.4-18.6]	**0.025**‡
Incident carpal tunnel syndrome (CTS)	460 (3.8%)	43 (3.1%)	29 (4.0%)	0.42
Incident ulnar nerve entrapment at elbow/wrist (UNE)	84 (0.7%)	16 (1.1%)	11 (1.5%)	**0.012**‡
Incident Dupuytren's contracture	245 (2.0)	32 (2.3%)	18 (2.5%)	0.56

Incident trigger finger	399 (3.3%)	41 (2.9%)	34 (4.7%)	0.091
Incident osteoarthritis of the thumb CMC-1 joint	297 (2.4%)	33 (2.4%)	17 (2.3%)	0.98
Any incident hand disorder	1,235 (10.1%)	135 (9.7%)	87 (11.9%)	0.24

P-value for good comparisons between the subjects are based on Kruskal Wallis and Mann-Whitney U-test as a posthoc test and Chi2 test for ordinal variables. † Statistical significance was found between "not at all" and "some". ‡ Statistical significance was found between "not at all" and "much". § Statistical significance found between "some" and "much".
