# Supplementary material for: Risk of hand and forearm conditions due to vibrating hand-held tools exposure: a retrospective cohort study from Sweden
Source: BMJ Open. 2024 Jun 18;14(6):e080777. doi: 10.1136/bmjopen-2023-080777 (PMC11191761; doi:10.1136/bmjopen-2023-080777)
Supplement: Author's manuscript [file bmjopen-2023-080777.draft_revisions.pdf]

BMJ Open

BMJ Open is committed to open peer review. As part of this commitment we make the peer review history of every article we publish publicly available.

When an article is published we post the peer reviewers' comments and the authors' responses online. We also post the versions of the paper that were used during peer review. These are the versions that the peer review comments apply to.

The versions of the paper that follow are the versions that were submitted during the peer review process. They are not the versions of record or the final published versions. They should not be cited or distributed as the published version of this manuscript.

BMJ Open is an open access journal and the full, final, typeset and author-corrected version of record of the manuscript is available on our site with no access controls, subscription charges or pay-per-view fees (http://bmjopen.bmj.com).

If you have any questions on BMJ Open's open peer review process please email info.bmjopen@bmj.com

Hand complications by exposure to vibrating hand-held tools – a retrospective cohort study

Journal:	*BMJ Open*
Manuscript ID	bmjopen-2023-080777
Article Type:	Original research
Date Submitted by the Author:	10-Oct-2023

Complete List of Authors:	Dahlin, Lars; Lund University, Department of Translational Medicine; Skånes universitetssjukhus Malmö, Department of Hand Surgery<br>Zimmerman, Malin; Lund University, Department of Translational Medicine – Hand Surgery; Helsingborgs lasarett, Department of Orthopedics<br>Nilsson, Peter; Lund University, Clinical Sciences<br>Rydberg, Mattias; Lund University, Department of Translational Medicine; Skånes universitetssjukhus Malmö, Department of Hand Surgery

Keywords:	Hand & wrist < ORTHOPAEDIC & TRAUMA SURGERY, Musculoskeletal disorders < ORTHOPAEDIC & TRAUMA SURGERY, General diabetes < DIABETES & ENDOCRINOLOGY, Hypertension < CARDIOLOGY, Aged

SCHOLARONE™
Manuscripts

I, the Submitting Author has the right to grant and does grant on behalf of all authors of the Work (as defined in the below author licence), an exclusive licence and/or a non-exclusive licence for contributions from authors who are: i) UK Crown employees; ii) where BMJ has agreed a CC-BY licence shall apply, and/or iii) in accordance with the terms applicable for US Federal Government officers or employees acting as part of their official duties; on a worldwide, perpetual, irrevocable, royalty-free basis to BMJ Publishing Group Ltd ("BMJ") its licensees and where the relevant Journal is co-owned by BMJ to the co-owners of the Journal, to publish the Work in this journal and any other BMJ products and to exploit all rights, as set out in our licence.

The Submitting Author accepts and understands that any supply made under these terms is made by BMJ to the Submitting Author unless you are acting as an employee on behalf of your employer or a postgraduate student of an affiliated institution which is paying any applicable article publishing charge ("APC") for Open Access articles. Where the Submitting Author wishes to make the Work available on an Open Access basis (and intends to pay the relevant APC), the terms of reuse of such Open Access shall be governed by a Creative Commons licence – details of these licences and which Creative Commons licence will apply to this Work are set out in our licence referred to above.

Other than as permitted in any relevant BMJ Author's Self Archiving Policies, I confirm this Work has not been accepted for publication elsewhere, is not being considered for publication elsewhere and does not duplicate material already published. I confirm all authors consent to publication of this Work and authorise the granting of this licence.

For peer review only

Hand complications by exposure to vibrating hand-held tools – a retrospective cohort study

Malin Zimmerman, MD, PhD, Assoc. Prof. [1,2,3], Peter M. Nilsson, MD, PhD, Prof. [4], Mattias Rydberg, MD, PhD [1,3], Lars B. Dahlin, MD, PhD, Prof [1,3,5*]

[1] Department of Translational Medicine – Hand Surgery, Lund University, Malmö, Sweden

[2] Department of Orthopedics, Helsingborg's Hospital, Helsingborg, Sweden

[3] Department of Hand Surgery, Skåne University Hospital, Malmö, Sweden

[4] Department of Clinical Sciences Malmö, Lund University, Malmö, Sweden

[5] Department of Biomedical and Clinical Sciences, Linköping University, Linköping, Sweden

* Corresponding author:

Lars B. Dahlin, Department of Translational Medicine – Hand Surgery, Lund University, Jan Waldenströms gata 5, 205 02 Malmö, Sweden; Fax +46 40 928855; lars.dahlin@med.lu.se

Word count: 2196

Keywords: carpal tunnel syndrome – osteoarthritis (OA) – Dupuytren's contracture – trigger finger disorder – ulnar nerve compression syndromes – vibration

For peer review only

Abstract

Objectives: The occurrence of hand disorders related to vibration exposure, adjusted for relevant background factors, is scarcely reported. We analyzed the prevalence of such conditions in a large population cohort, stratified by sex, and associations with exposure to vibrating hand-held tools.

Methods: Individuals in the Malmö Diet and Cancer Study cohort (inclusion 1991-1996; followed until 2018) were asked, *"Does your work involve working with vibrating hand-held tools?"* (response: "Not at all", "Some" and "Much"). Data was cross-linked with national registers to identify treatment for carpal tunnel syndrome (CTS), ulnar nerve entrapment (UNE), Dupuytren's disease, trigger finger, or CMC-1osteoarthritis (OA). Cox regression models, unadjusted and adjusted (age, sex, prevalent diabetes, smoking, hypertension, and alcohol consumption), were performed to analyze the effects of reported vibration exposure.

Results: In total, 12,220/16,104 individuals (76%) reported no exposure, 1,392/16,104 (9%) "some" and 730/16,104 (5%) "much" exposure to vibrating hand-held tools. In men, much exposure was independently associated with CTS [HR 1.71 (95% CI 1.11-2.62)] and UNE [HR 2.42 (1.15-5.07)]. Some exposure was independently associated with UNE in men [HR 2.10 (1.12-3.95)]. Much exposure was independently associated with trigger finger in women [2.73 (1.49-4.99)]. We found no effect of vibration exposure on Dupuytren's disease or CMC-1 OA. Much vibration exposure predicted any hand diagnosis in men [HR 1.44 (1.08-1.80)] but not in women.

For peer review only - http://bmjopen.bmj.com/site/about/guidelines.xhtml

Conclusions: Vibration exposure by hand-held tools increases the risk of developing CTS and UNE and any common hand complication in men, whereas women risk trigger finger. Adjustment for relevant confounders in vibration exposure is crucial.

Strengths and limitations of this study:

- The main strength is that different hand complications, with long-term follow-up, were analyzed in the same population-based cohort of men and women, with calculated hazard ratios (HR) adjusted for relevant confounders.
- Quantification of vibration exposure is not possible in larger cohorts, but the used simple grading (i.e., “no”, “some”, and “much”) showed an increased risk for some hand complications.
- A general 41% response rate was obtained among the population-based cohort, with no information on vibration exposure in >10 % of the individuals.
- Diagnoses were based on ICD-codes from the National Board of Health and Welfare, including both surgically and not surgically treated cases, but with no individuals from primary care.

For peer review only

INTRODUCTION

Hand-arm vibration syndrome (HAVS) consists of symptoms and functional, social, emotional, and psychological disability from injuries to various tissue components of the upper extremity, such as muscle, nerve, connective tissue, and joints.[1-4] Vibration exposure is reported to induce pathological conditions, including carpal tunnel syndrome (CTS),[5 6] ulnar nerve entrapment at the wrist or the elbow (UNE),[7] Dupuytren's contracture,[8] or osteoarthritis of the first carpometacarpal joint (CMC-1),[7] all of which may severely affect the patient's hand function and ability to work. The most well-known symptoms are related to the blood vessels, where vibration exposure provokes vibration-induced white fingers (VWF).[9 10] Muscle weakness is a clinically common symptom that is described by individuals exposed to hand-held vibrating tools, and structural changes in the muscles have been described both in experimental studies and in human muscle biopsies.[11] In addition, nerve biopsies from the posterior interosseous nerve just proximal to the wrist from patients exposed to vibrating hand-held tools indicate that vibration exposure induces neuropathy with substantial axonal loss.[12] Such a neuropathy may be the underlying reason why exposed patients experience CTS-like symptoms or have a propensity to develop CTS.[3] An increased occurrence of osteoarthritis in joints of the hand related to vibration exposure has been suggested.[13] A less highlighted condition, although questioned to be work-related,[14] and linked to exposure to vibrating hand-held tools is Dupuytren's disease with finger contracture affecting the palmar fascia and bending of fingers.[8] However, a genetic component[15] as well as a relation to diabetes and occupation[16] may be more common causes of Dupuytren´s disease. Vibration exposure may promote or speed up the development of Dupuytren's contracture in a genetically predisposed individual. Most previous studies report on one of the hand complications, and few have reported the occurrence of all conditions suggested to be related to vibration exposure.

For peer review only - http://bmjopen.bmj.com/site/about/guidelines.xhtml

We aimed to analyze the presence of five common hand disorders in a large population-based cohort, where a proportion of the individuals reported that they were "not at all" exposed or exposed to vibrating hand-held tools to "some" or "much" extent.

METHODS

Study population

A sizeable population-based register from southern Sweden, the Malmö Diet and Cancer Study (MDCS), which has previously been described,[17 18] was used in this observational study. In brief, 30,446 individuals aged 46-73 were invited to participate between 1991-1996. Initially, individuals were screened for cardiovascular risk factors, undergoing clinical examination and laboratory assessment under fasting conditions. In addition, they responded to several questionnaires. One of the questionnaires contained the question, *"Does your work involve working with vibrating hand-held tools?"* where the individuals responded, *"Not at all"*, *"Some"* and *"Much"*. This questionnaire was answered by 14,342 individuals included in the present study. In addition, other relevant variables were noted, including smoking, alcohol consumption, hypertension, and diabetes mellitus. Smoking was self-reported at baseline. Alcohol consumption was self-reported use during the last week at baseline and reported as g/day. Diabetes data was obtained to register linkage with the National Diabetes Register (NDR), which contains data from both hospital care and primary health care on patients with diabetes.

Endpoint retrieval

In the present study, we used data from national registers managed by the National Board of Health and Welfare to identify whether the included individuals had been treated for the hand conditions of interest. The used registers include data from hospitals and specialized care

For peer review only - http://bmjopen.bmj.com/site/about/guidelines.xhtml

settings (outpatient clinics) but not from primary care units. Inpatient care has been registered since 1964, outpatient surgeries are included from 1997, and visits in specialized outpatient clinics are included from 2001. The included hand conditions were the two most common nerve compression disorders [carpal tunnel syndrome (ICD-10 code G56.0) and ulnar nerve entrapment at the elbow or the wrist (G56.2)], Dupuytren's disease with contracture (M72.0), trigger finger (M65.3) and osteoarthritis of the first CMC joint (M18.X). Patients were followed until death, emigration, or end of study 2018-12-31, whichever occurred first.

Statistical analysis

The baseline characteristics are presented as median (25th to 75th percentiles) or proportion of patients n (%). The Kruskal-Wallis test was used to detect any statistical significance. If p-value <0.05, a Mann-Whitney U-test was used to identify individual significant differences between the three groups. For ordinal variables, the Chi^2-test was used for group comparisons. Cox regression analysis was performed to analyze the effect of vibration exposure on the development of hand pathology. In the group with much vibration exposure, there were 82% men, so we chose to present the results stratified by sex. The group without vibration exposure was used as a reference group. The first models were unadjusted. The second models were adjusted for age at baseline, sex, prevalent diabetes before the study started, smoking, hypertension, and alcohol consumption based on previous data and on clinical experience on relevant influencing factors for the specific diagnosis[19-21] (see also Discussion). Kaplan Meier curves were used to visualize disease-free survival. All statistical analysis was made using IBM SPSS Statistics for Mac version 28 (SPSS Inc., Chicago, IL, USA), and a two-sided p-value <0.05 was considered significant.

Patient and Public Involvement Statement

For peer review only - http://bmjopen.bmj.com/site/about/guidelines.xhtml

No patients, politicians, or the public were involved in elaborating on the present research question or the design of the study. The obtained results, after publication in the scientific journal, will be disseminated in different ways. Information will be provided to physicians and surgeons working with patients diagnosed with HAVS in their everyday practice. This also includes the healthcare organizations connected to small and large companies. Other colleagues and students in the healthcare sector and any patient organizations or insurance companies interested in HAVS will also be included in information policies.

RESULTS

Characteristics

The baseline characteristics are presented in **Table 1**. In total, 12,220/16,104 individuals (76%) reported that they did not have had any exposure to vibrating hand-held tools, while 1,392/16,104 (9%) reported that they had "some" and 730/16,104 (5%) had "much" exposure to vibrating hand-held tools. Some differences in basic characteristics were observed among men and women related to the extent of reported vibration exposure.

Table 1. Baseline characteristics of all included individuals that reported exposure to vibrating hand-held tools as "not at all", "some" and "much".

	"Does your work involve working with vibrating hand-held tools?"			
	**Not at all**	**Some**	**Much**	**P-value**
	**12,220 (85%)**	**1,392 (10%)**	**730 (5%)**	

For peer review only - http://bmjopen.bmj.com/site/about/guidelines.xhtml

For peer review only

Age (years)	57±6	57±6	57±6	0.18
Sex (men)	4845 (40%)	1003 (72%)	601 (82%)	**<0.001**†,‡,§
Current smoking	3458 (28%)	477 (34%)	257 (35%)	**<0.001**†,‡
Antihypertensive treatment	5763 (47%)	708 (51%)	373 (51%)	**0.005**†,‡
Prevalent diabetes	546 (5%)	85 (6%)	37 (5%)	**0.02**†
Alcohol consumption (g/day)	7.3 [1.7-15.5]	8.1 [1.8-17.3]	9.0 [1.4-18.6]	**0.025**‡
Incident carpal tunnel syndrome (CTS)	460 (3.8%)	43 (3.1%)	29 (4.0%)	0.42
Incident ulnar nerve entrapment at elbow/wrist (UNE)	84 (0.7%)	16 (1.1%)	11 (1.5%)	**0.012**‡
Incident Dupuytren's contracture	245 (2.0)	32 (2.3%)	18 (2.5%)	0.56
Incident trigger finger	399 (3.3%)	41 (2.9%)	34 (4.7%)	0.091
Incident osteoarthritis of the thumb CMC-1 joint	297 (2.4%)	33 (2.4%)	17 (2.3%)	0.98

For peer review only - http://bmjopen.bmj.com/site/about/guidelines.xhtml

Any incident hand disorder	1,235 (10.1%)	135 (9.7%)	87 (11.9%)	0.24

P-value for good comparisons between the subjects are based on Kruskal Wallis and Mann-Whitney U-test as a posthoc test and Chi2 test for ordinal variables. † Statistical significance was found between "not at all" and "some". ‡ Statistical significance was found between "not at all" and "much". § Statistical significance found between "some" and "much".

Carpal tunnel syndrome and ulnar nerve compression at the elbow or wrist

Among the men, CTS and UNE were more common in the groups with more vibration exposure; however, not being statistically significant for CTS (**Table 2**). In the Cox regression analysis, much vibration exposure was independently associated with CTS [Hazard Ratio 1.71 (95% CI 1.11-2.62] and UNE [HR 2.42 (1.15-5.07); **Table 3; Figure 1A and C**]. Some vibration exposure was independently associated with UNE [HR 2.10 (1.12-3.95); **Table 3; Figure 1C**].

Among the women, CTS seemed more common in the group without vibration exposure, however, it was not statistically significant. The Cox regression models for women showed no significant effects of vibration exposure on the risk of developing CTS and UNE (**Figure 1B and D**).

For peer review only

Table 2. Baseline characteristics of individuals that reported exposure to vibrating hand-held tools as "not at all", "some" and "much" stratified by sex.

	"Does your work involve working with vibrating hand-held tools?"							
	**Men (n=6448)**				**Women (n=7892)**			
	**Not at all n=4,845**	**Some n=1,003**	**Much n=601**	**P-value**	**Not at all n=7,374**	**Some n=389**	**Much n=129**	**P-value**
Age (years)	57±6	57±6	57±6	0.67	57±6	58±6	57±6	0.079
Current smoking	1,355 (28%)	355 (35%)	211 (35%)	**<0.001**†,‡	2,103 (29%)	122 (31%)	46 (36%)	0.10
Antihypertensive treatment	2,499 (52%)	529 (53%)	329 (55%)	0.306	3,264 (44%)	179 (46%)	44 (34%)	0.053
Prevalent diabetes	269 (6%)	69 (7%)	33 (6%)	0.249	277 (4%)	16 (4%)	4 (3%)	0.87
Alcohol consumption (g/day)	12.2 [4.5-23.0]	9.6 [2.9-21.3]	10.6 [1.8-20.1]	**<0.001**†,‡	5.4 [0.8-11.2]	4.4 [0.46-10.0]	4.1 [0.0-10.3]	**0.019**†
Incident carpal tunnel syndrome (CTS)	133 (2.7%)	34 (3.4%)	26 (4.3%)	0.072	327 (4.4%)	9 (2.3%)	3 (2.3%)	0.071

For peer review only - http://bmjopen.bmj.com/site/about/guidelines.xhtml

Incident ulnar nerve entrapment at elbow/wrist (UNE)	33 (0.7%)	14 (1.4%)	9 (1.5%)	**0.019**‡	51 (0.7%)	2 (0.5%)	2 (1.6%)	0.46
Incident Dupuytren's contracture	158 (3.3)	25 (2.5%)	16 (2.7%)	0.36	87 (1.2)	7 (1.8%)	2 (1.6%)	0.52
Incident trigger finger	137 (2.8%)	28 (2.8%)	23 (3.8%)	0.38	262 (3.6%)	13 (3.3%)	11 (8.5%)	**0.011**‡,§
Incident osteoarthritis of the thumb CMC-1 joint	61 (1.3%)	19 (1.9%)	11 (1.8%)	0.20	236 (3.2%)	14 (3.6%)	6 (4.7%)	0.60
Any incident hand disorder	442 (9.1%)	94 (9.4%)	69 (11.5%)	0.17	793 (10.8%)	41 (10.5%)	18 (14%)	0.50

P-value for good comparisons between the subjects are based on Kruskal Wallis and Mann-Whitney U-test as a posthoc test and Chi2 test for ordinal variables. † Statistical significance was found between "not at all" and "some". ‡ Statistical significance was found between "not at all" and "much". § Statistical significance found between "some" and "much".

For peer review only - http://bmjopen.bmj.com/site/about/guidelines.xhtml

Table 3. Cox regression models – unadjusted and adjusted for relevant factors – with the association between vibration exposure and risk for development of five specific hand disorders and any of the hand disorders stratified by sex.

**Men**						
Model 1 - unadjusted						
	**CTS**	**UNE**	**Trigger finger**	**Dupuytren's contracture**	**CMC-1 osteoarthritis**	**Any hand disorder**
No vibration exposure	*Reference*					
Some vibration exposure	1.21 (0.82-1.79)	2.20 (1.17-4.12)	1.01 (0.67-1.53)	0.78 (0.51-1.20)	1.57 (0.94-2.64)	1.05 (0.84-1.32)
Much vibration exposure	1.71 (1.11-2.62)	2.44 (1.17-5.12)	1.50 (0.97-2.34)	0.90 (0.54-1.50)	1.55 (0.82-2.95)	1.40 (1.08-1.80)
Model 2 – adjusted for age, smoking, alcohol consumption, hypertension and diabetes						
No vibration exposure	Reference					
Some vibration exposure	1.18 (0.80-1.76)	2.10 (1.12-3.95)	1.01 (0.67-1.53)	0.82 (0.53-1.27)	1.55 (0.92-2.61)	1.06 (0.84-1.33)
Much vibration exposure	1.71 (1.11-2.62)	2.42 (1.15-5.07)	1.54 (0.99-2.39)	0.96 (0.57-1.61)	1.55 (0.81-2.94)	1.44 (1.12-1.86)

**Women**						
Model 1 - unadjusted						
	**CTS**	**UNE**	**Trigger finger**	**Dupuytren's contracture**	**CMC-1 osteoarthritis**	**Any hand disorder**
No vibration exposure	*Reference*					
Some vibration exposure	0.56 (0.29-1.08)	0.83 (0.20-3.40)	1.02 (0.59-1.78)	1.67 (0.77-3.60)	1.20 (0.70-2.06)	1.06 (0.77-1.45)

For peer review only - http://bmjopen.bmj.com/site/about/guidelines.xhtml

Much vibration exposure	0.56 (0.18-1.75)	2.50 (0.61-10.26)	2.72 (1.49-4.96)	1.43 (0.35-5.83)	1.55 (0.69-3.48)	1.43 (0.89-2.28)
Model 2 – adjusted for age, smoking, alcohol consumption, hypertension, and diabetes						
No vibration exposure	Reference					
Some vibration exposure	0.56 (0.29-1.09)	0.82 (0.20-3.36)	1.05 (0.60-1.83)	1.76 (0.81-3.80)	1.19 (0.69-2.03)	1.07 (0.78-1.46)
Much vibration exposure	0.56 (0.18-1.76)	2.54 (0.62-10.48)	2.73 (1.49-4.99)	1.57 (0.39-6.38)	1.57 (0.70-3.54)	1.45 (0.91-2.32)

CTS; carpal tunnel syndrome, UNE; ulnar nerve entrapment at elbow/wrist, CMC-1; first carpometacarpal joint

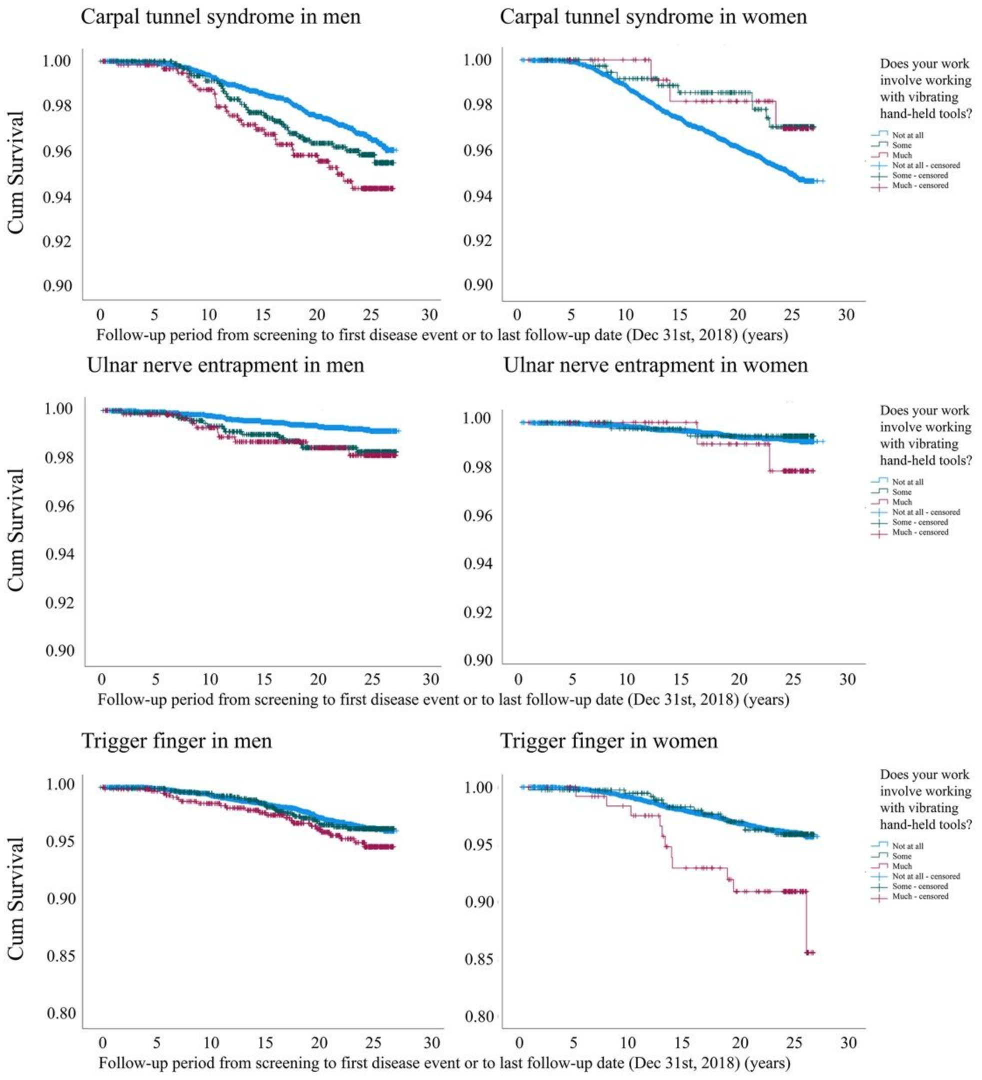

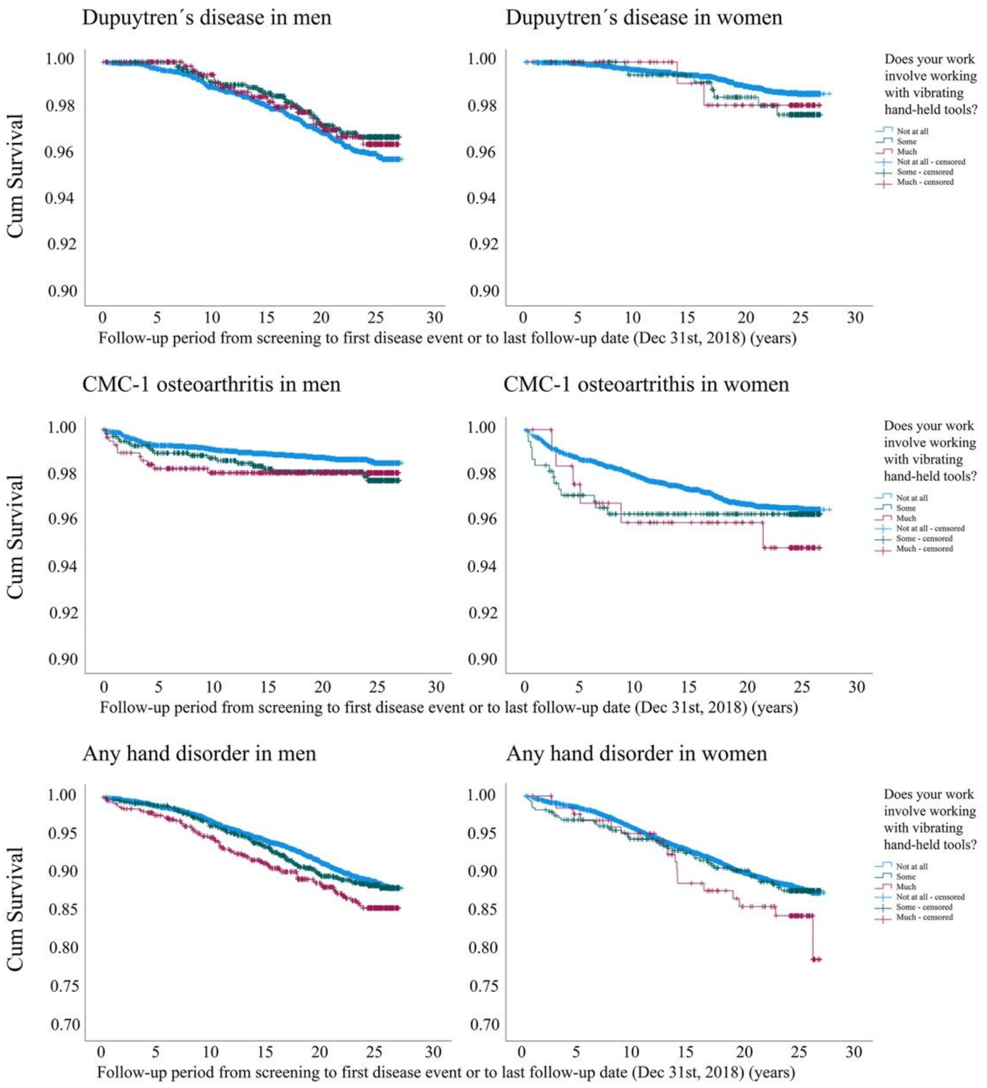

Figure 1. Survival analysis using Kaplan Meier curves of the treated hand disorders that were followed from screening to the first disease event or to the last follow-up date (Dec 31[st,] 2018) and were potentially associated with vibration exposure stratified by sex. The individuals replied to the question "Does your work involve working with vibrating hand-held tools?" with possible answers "Not at all", "Some" and "Much".

Trigger finger

Trigger finger was more common among women with much vibration exposure than women with some or no vibration exposure (**Table 2**). No differences could be seen between the groups of men **[Figure 1E]**. Much vibration exposure was independently associated with the

For peer review only - http://bmjopen.bmj.com/site/about/guidelines.xhtml

development of trigger finger in women [HR 2.73 (1.49-4.99)], but not in men (**Table 3; Figure 1E and F**).

Dupuytren's contracture

Dupuytren's contracture was equally common among the groups in both sexes, and no significant effect of vibration exposure on the development of Dupuytren's disease with contracture could be demonstrated in the Cox regression analysis (**Table 2** and **3; Figure 1G and H**).

Osteoarthritis of the first CMC joint

CMC-1 OA was equally common among the groups in both sexes, and no significant effect of vibration exposure on the development of CMC-1 OA could be demonstrated in the Cox regression analysis (**Table 2** and **3; Figure 1I and J**).

Any hand diagnosis

We found similar proportions of any incident hand diagnosis among all groups (**Tables 1 and 2**). In the Cox regression analysis, much vibration exposure predicted any hand diagnosis in men [HR 1.44 (1.12-1.86)] but not in women [HR 1.45 (0.91-2.32)] (**Table 3; Figure 1K and L**).

DISCUSSION

The present observational study of a large population-based cohort, where the individuals replied to a question concerning exposure to vibrating hand-held tools and graded as "not at all", "some" and "much", shows that hand complications in men, such as CTS and UNE, were significantly higher than in un-exposed men, particularly among those with "much" vibration

For peer review only

exposure when adjusted for several relevant confounding factors. In addition, any hand complication was associated with “much” exposure to vibrating hand-held tools. In contrast, in women, there were no associations between vibration and the described hand disorders, except an association between “much” vibration exposure and trigger finger. The presently described hand conditions are common in the general population, irrespective of vibration exposure, where prevalence figures for CTS, UNE, Dupuytren´s contracture, trigger finger, and osteoarthritis of the CMC-1 joint are reported as 1.4-15 %.[20 22 23] Previously published data has mainly included one of the present diagnoses at a time associated to vibration exposure, while the data from the current cohort also allowed the inclusion of all the relevant, and previously discussed, conditions in the hand associated to exposure to vibrating hand-held tools. [8 24-26]

Risk assessment in vibration exposure of hand-held tools to different hand disorders may be complex due to the high prevalence of some of the hand disorders as well as the association with relevant factors and general comorbidities that must be adjusted for in any statistical analysis.[8 13 21 26 27] In this study, we stratified our data on sex due to the relatively high number of male individuals in the cohort, as well attempting to level out any difference in occurrence of vibration exposure in men and women as sex is known to influence the prevalence of hand diagnoses.[28] In our *Model 2*, we adjusted for factors that may be of relevance in development of the included hand disorders, i.e., age, smoking, alcohol consumption, hypertension, and diabetes; variables that are accessible in the MDCS database.[19 27] Age is a highly relevant influencing factor for development hand disorders, where men and women have different age distributions for development of CTS and UNE as well as of osteoarthritis in the first CMC joint.[23 28 29] In contrast to our study, other authors have reported an increased risk for CTS in both sexes, although being highest among young men,[24] which may be explained by

For peer review only - http://bmjopen.bmj.com/site/about/guidelines.xhtml

differences between the cohorts and adjustment of confounding factors. Smoking may affect both the development and outcome of surgery of CTS and UNE,[27] due to its effect on the intraneural microcirculation as well as being highlighted in Dupuytren´s disease with contracture.[30] Individuals with hypertension may also have a higher perfusion pressure in the intraneural blood vessels making the nerve more resistant to compression [31]; again, indicating that adjustment for several factors in the Cox analysis is necessary.[32] Development of CTS, UNE, Dupuytren´s disease with finger contracture, and trigger fingers are also associated with both type 1 and type 2 diabetes,[19] which is probably not entirely true for osteoarthritis of the first CMC joint, for which high BMI and obesity are significant risk markers/factors.[33] Finally, alcohol consumption is also a factor that may be involved in development of Dupuytren´s contracture as well as a cause of UNE.[19 34] Thus, it is crucial to adjust for age, smoking, alcohol consumption, hypertension and diabetes when analyzing the risk of developing various hand disorders in relation to vibration exposure of hand-held tools. We also included the association of any hand disorder as a variable in relation to vibration exposure, which is appropriate since there usually is an extensive co-morbidity of various hand disorders, such as concomitant CTS in surgically treated patients with UNE, as well as the other present hand conditions.[35] In addition, a subject with diabetes, irrespective of having type 1 or type 2 diabetes or if neuropathy is present, may have an increased susceptibility to a nerve compression disorders as a subject with a history of vibration exposure.

The observed differences in risks for developing the studied hand conditions in men and women are exciting and may have different explanations. Surprisingly, in women, much vibration exposure only increased the risk of trigger finger, while in men, the risks were higher for CTS and UNE. There are no apparent reasons for this discrepancy. Still, it has been suggested that an underlying neuropathy may be important for development of CTS in

For peer review only - http://bmjopen.bmj.com/site/about/guidelines.xhtml

vibration-exposed individuals.[3 8] One other study, however, found that working with vibrating tools more than two hours per day was a risk factor for developing CTS in women, but not in men.[36] Furthermore, neuropathy is reported to be more common and more severe in men with diabetes.[24 37] Therefore, one may speculate that similar mechanisms may explain the present sex difference concerning the nerve compression disorders. Trigger finger is more common among women, and it has been speculated that estrogen deficiency alters tendon metabolism in postmenopausal women, hence impacting the risk of developing trigger finger.[20] Previous studies have failed to demonstrate an association between occupational factors and trigger finger.[20] It is, however, possible that during the early 1990s, when this cohort study started, occupational sex differences of risk existed. It is possible that among the participants who worked with vibrating hand-held tools, the men worked with large tools, such as grinding machines and chainsaws. In contrast, women may have worked more with precision dental drills and other smaller tools. Whether the type of tool affects these disorders' development is unknown.

Our data also indicates that there is no greater risk of osteoarthritis in the first CMC joint in vibration-exposed individuals, indicating that the development of such a condition has other causes than vibration exposure alone.[25]

It was not possible and was not the present study's purpose to evaluate any surgery outcome for the conditions in the individuals with no, some, or much exposure to vibration. The result of surgery in vibration-exposed patients with CTS seems to be impaired compared to otherwise healthy patients with CTS,[38]. Still, outcome data are essentially lacking for the other hand complications included in the present study, except for Dupuytren´s contracture, for which socioeconomic status has to be considered.[39] An earlier exposure to vibrating tools

For peer review only - http://bmjopen.bmj.com/site/about/guidelines.xhtml

may affect the pre- and postoperative function in individuals undergoing surgery for Dupuytren's contracture. However, the satisfaction or return to work was not different.[40]

Limitations

There are some limitations of the study, such as that in >10 % of the individuals, no information was available concerning vibration exposure as well as a general 41% response rate among the population-based cohort.[18] In addition, our diagnoses were based on ICD-codes from the National Board of Health and Welfare, where both surgically and not surgically treated cases were included, although without any patients diagnosed in primary care. Quantifying the vibration exposure is relevant but is not possible in larger cohorts as in the present study, which is a limitation. However, the simple grading in no, some, and much indicated an increased risk for some hand complications.

CONCLUSIONS

We conclude that vibration exposure to hand-held tools increases the risk of having or developing CTS, and particularly UNE, but also any common hand complication in men and trigger finger in women. It is essential to adjust for relevant influencing factors, such as age, smoking, alcohol consumption, hypertension, and diabetes, when investigating the risks of working with hand-held vibrating tools.

STATEMENTS

Author contributions

M.Z., P.N., and L.B.D. conceived the ideas; M.Z. and P.N. collected the data; M.Z., M.R., and L.B.D. analyzed the data; M.Z. and L.B.D. led the writing, and all authors contributed to the interpretation and the writing and approved the final version of the manuscript.

For peer review only - http://bmjopen.bmj.com/site/about/guidelines.xhtml

Funding statement

This research was funded by the Swedish Research Council (#2022-01942, principal investigator LBD), the Swedish Diabetes Foundation (#DIA2020-492, principal investigator LBD), the Regional Agreement on Medical Training and Clinical Research (ALF; PI LBD) between Region Skåne and Lund University (#2018-Projekt 0104; PI LBD) and Funds from Skåne University Hospital (#2019-659; PI LBD), Elly Olsson´s Foundation for scientific research (#N/A), Stig and Ragna Gorthon Foundation (#N/A), Almroth Foundation (#N/A), Kockska foundation (#N/A), and the Magnus Bergvall Foundation [2020-03612, principal investigator MZ].

The initial financial support to the Malmö Diet and Cancer Study cohort at baseline was provided by the Swedish Cancer Foundation (#N/A), The Swedish Medical Research Council (#N/A), The European Commission (#N/A), the City of Malmö (#N/A), the Swedish Dairy Association (#N/A) and the Albert Påhlsson Foundation (#N/A).

The sponsors had no role in the study design, collection, analysis, or interpretation of the data, writing the report, or decision to submit the manuscript.

Competing of Interests

The authors declare no conflict of interest.

Ethics Approval

Participants provided written informed consent before inclusion in MDCS. MDCS and the present study have been approved by the regional Ethics committee in Lund and the Swedish Ethical Review Authority (DNR: LU51-90; 2009-633; 2019-01439). The study was conducted in accordance with the Helsinki Declaration.

For peer review only - http://bmjopen.bmj.com/site/about/guidelines.xhtml

Data Sharing

The data are not publicly available due to ethical restrictions. The data that support the findings of this study are only available on request from the corresponding author as well as after application and approval by the Swedish Ethical Review Authority (https://etikprovningsmyndigheten.se/en/ due to the restrictions in Sweden, based on the national Law for ethical review of research on humans [“Lag (2003:460) om etikprövning av forskning som avser människor”].

For peer review only

Acknowledgments

The authors are very grateful to all the participants in MDCS whose participation enabled this work. We are also thankful to the National Diabetes Register (NDR) and Statistics Sweden (SCB) for register data, Anders Dahlin for his help with data extraction, and Tina Folker for her administrative assistance. We acknowledge support from the Lund University Infrastructure grant ”Malmö population-based cohorts” (STYR 2019/2046).

References

1. Dong RG, Rakheja S, Schopper AW, et al. Hand-transmitted vibration and biodynamic response of the human hand-arm: a critical review. *Crit Rev Biomed Eng* 2001;29(4):393-439. doi: 10.1615/critrevbiomedeng.v29.i4.20

For peer review only - http://bmjopen.bmj.com/site/about/guidelines.xhtml

2. Pelmear P, Leong D. Review of occupational standards and guidelines for hand-arm (segmental) vibration syndrome (HAVS). *Appl Occup Environ Hyg* 2000;15(3):291-302.

3. Dahlin LB, Sanden H, Dahlin E, et al. Low myelinated nerve-fibre density may lead to symptoms associated with nerve entrapment in vibration-induced neuropathy. *J Occup Med Toxicol* 2014;9(1):7. doi: 10.1186/1745-6673-9-7

4. Lundborg G, Dahlin LB, Hansson H-A, et al. Vibration exposure and peripheral nerve fiber damage. *J Hand Surg* 1990;15A:346-51.

5. Hulkkonen S, Shiri R, Auvinen J, et al. Risk factors of hospitalization for carpal tunnel syndrome among the general working population. *Scand J Work Environ Health* 2020;46(1):43-49. doi: 10.5271/sjweh.3835 [published Online First: 20190606]

6. Lawson IJ. Is carpal tunnel syndrome caused by work with vibrating tools? *Occup Med (Lond)* 2020;70(1):8-10. doi: 10.1093/occmed/kqz142

7. Piligian G, Herbert R, Hearns M, et al. Evaluation and management of chronic work-related musculoskeletal disorders of the distal upper extremity. *Am J Ind Med* 2000;37(1):75-93. doi: 10.1002/(sici)1097-0274(200001)37:1<75::aid-ajim7>3.0.co;2-4

8. Mathieu S, Naughton G, Descatha A, et al. Dupuytren's Disease and exposure to vibration: Systematic review and Meta-analysis. *Joint Bone Spine* 2020;87(3):203-07. doi: 10.1016/j.jbspin.2020.02.001 [published Online First: 20200212]

9. Aarhus L, Stranden E, Nordby KC, et al. Vascular component of hand-arm vibration syndrome: a 22-year follow-up study. *Occup Med (Lond)* 2018;68(6):384-90. doi: 10.1093/occmed/kqy085

10. Wei N, Yan R, Lang L, et al. Local vibration induced vascular pathological structural changes and abnormal levels of vascular damage indicators. *Microvasc Res*

For peer review only - http://bmjopen.bmj.com/site/about/guidelines.xhtml

2021;136:104163. doi: 10.1016/j.mvr.2021.104163 [published Online First: 20210406]

11. Necking LE, Lundborg G, Lundstrom R, et al. Hand muscle pathology after long-term vibration exposure. *The Journal of Hand Surgery British Volume* 2004;29(5):431-7. doi: 10.1016/j.jhsb.2004.05.004

12. Stromberg T, Dahlin LB, Brun A, et al. Structural nerve changes at wrist level in workers exposed to vibration. *Occup Environ Med* 1997;54(5):307-11.

13. Hammer PE, Shiri R, Kryger AI, et al. Associations of work activities requiring pinch or hand grip or exposure to hand-arm vibration with finger and wrist osteoarthritis: a meta-analysis. *Scand J Work Environ Health* 2014;40(2):133-45. doi: 10.5271/sjweh.3409 [published Online First: 2013/12/07]

14. Descatha A, Jauffret P, Chastang JF, et al. Should we consider Dupuytren's contracture as work-related? A review and meta-analysis of an old debate. *BMC Musculoskelet Disord* 2011;12:96. doi: 10.1186/1471-2474-12-96 [published Online First: 2011/05/18]

15. Ng M, Thakkar D, Southam L, et al. A Genome-wide Association Study of Dupuytren Disease Reveals 17 Additional Variants Implicated in Fibrosis. *Am J Hum Genet* 2017;101(3):417-27. doi: 10.1016/j.ajhg.2017.08.006

16. van den Berge BA, Wiberg A, Werker PMN, et al. Dupuytren's disease is a work-related disorder: results of a population-based cohort study. *Occup Environ Med* 2023;80(3):137-45. doi: 10.1136/oemed-2022-108670 [published Online First: 20230112]

17. Berglund G, Elmstahl S, Janzon L, et al. The Malmo Diet and Cancer Study. Design and feasibility. *J Intern Med* 1993;233(1):45-51. doi: 10.1111/j.1365-2796.1993.tb00647.x [published Online First: 1993/01/01]

For peer review only

18. Manjer J, Carlsson S, Elmstahl S, et al. The Malmo Diet and Cancer Study: representativity, cancer incidence and mortality in participants and non-participants. *Eur J Cancer Prev* 2001;10(6):489-99. doi: 10.1097/00008469-200112000-00003 [published Online First: 2002/03/28]

19. Rydberg M, Zimmerman M, Lofgren JP, et al. Metabolic factors and the risk of Dupuytren's disease: data from 30,000 individuals followed for over 20 years. *Sci Rep* 2021;11(1):14669. doi: 10.1038/s41598-021-94025-7 [published Online First: 2021/07/21]

20. Okita G, Hayashi M, Ikegami S, et al. The Prevalence and Risk Factors for Trigger Digits in a Random Sampling of a Japanese Population Registry between 50 and 89 Years of Age. *J Hand Surg Asian Pac Vol* 2022;27(1):148-55. doi: 10.1142/S2424835522500151 [published Online First: 20220209]

21. Jones G, Cooley HM, Stankovich JM. A cross sectional study of the association between sex, smoking, and other lifestyle factors and osteoarthritis of the hand. *J Rheumatol* 2002;29(8):1719-24.

22. Haara MM, Heliovaara M, Kroger H, et al. Osteoarthritis in the carpometacarpal joint of the thumb. Prevalence and associations with disability and mortality. *J Bone Joint Surg Am* 2004;86(7):1452-7. doi: 10.2106/00004623-200407000-00013

23. Atroshi I, Gummesson C, Johnsson R, et al. Prevalence of carpal tunnel syndrome in a general population [see comments]. *JAMA* 1999;282(2):153-58.

24. Vihlborg P, Pettersson H, Makdoumi K, et al. Carpal Tunnel Syndrome and Hand-Arm Vibration: A Swedish National Registry Case-Control Study. *J Occup Environ Med* 2022;64(3):197-201. doi: 10.1097/JOM.0000000000002451

For peer review only

25. Malchaire J, Maldague B, Huberlant JM, et al. Bone and joint changes in the wrists and elbows and their association with hand and arm vibration exposure. *Ann Occup Hyg* 1986;30(4):461-8. doi: 10.1093/annhyg/30.4.461

26. van Rijn RM, Huisstede BM, Koes BW, et al. Associations between work-related factors and the carpal tunnel syndrome--a systematic review. *Scand J Work Environ Health* 2009;35(1):19-36. doi: 10.5271/sjweh.1306

27. Pourmemari MH, Viikari-Juntura E, Shiri R. Smoking and carpal tunnel syndrome: a meta-analysis. *Muscle Nerve* 2014;49(3):345-50. doi: 10.1002/mus.23922 [published Online First: 20130708]

28. Rydberg M, Zimmerman M, Gottsater A, et al. Diabetic hand: prevalence and incidence of diabetic hand problems using data from 1.1 million inhabitants in southern Sweden. *BMJ Open Diabetes Res Care* 2022;10(1) doi: 10.1136/bmjdrc-2021-002614 [published Online First: 2022/01/21]

29. Mitake T, Iwatsuki K, Hirata H. Differences in characteristics of carpal tunnel syndrome between male and female patients. *J Orthop Sci* 2020;25(5):843-46. doi: 10.1016/j.jos.2019.10.017 [published Online First: 20191125]

30. Eckerdal D, Nivestam A, Dahlin LB. Surgical treatment of Dupuytren's disease - outcome and health economy in relation to smoking and diabetes. *BMC Musculoskelet Disord* 2014;15:117. doi: 10.1186/1471-2474-15-117 [published Online First: 20140402]

31. Szabo RM, Gelberman RH, Williamson RV, et al. Effects of increased systemic blood pressure on the tissue fluid pressure threshold of peripheral nerve. *J Orthop Res* 1983;1(2):172-8. doi: 10.1002/jor.1100010208

32. Mustafa KN, Khader YS, Bsoul AK, et al. Musculoskeletal disorders of the hand in type 2 diabetes mellitus: prevalence and its associated factors. *Int J Rheum Dis* 2016;19(7):730-5. doi: 10.1111/1756-185X.12617

For peer review only - http://bmjopen.bmj.com/site/about/guidelines.xhtml

33. Rydberg M, Dahlin LB, Gottsater A, et al. High body mass index is associated with increased risk for osteoarthritis of the first carpometacarpal joint during more than 30 years of follow-up. *RMD Open* 2020;6(3) doi: 10.1136/rmdopen-2020-001368 [published Online First: 2020/10/29]

34. Nordenskjold J, Englund M, Zhou C, et al. Prevalence and incidence of doctor-diagnosed Dupuytren's disease: a population-based study. *J Hand Surg Eur Vol* 2017;42(7):673-77. doi: 10.1177/1753193416687914

35. Giostad A, Nyman E. Patient Characteristics in Ulnar Nerve Compression at the Elbow at a Tertiary Referral Hospital and Predictive Factors for Outcomes of Simple Decompression versus Subcutaneous Transposition of the Ulnar Nerve. *Biomed Res Int* 2019;2019:5302462. doi: 10.1155/2019/5302462 [published Online First: 2020/01/18]

36. Melchior M, Roquelaure Y, Evanoff B, et al. Why are manual workers at high risk of upper limb disorders? The role of physical work factors in a random sample of workers in France (the Pays de la Loire study). *Occup Environ Med* 2006;63(11):754-61. doi: 10.1136/oem.2005.025122 [published Online First: 20060620]

37. Kamenov ZA, Parapunova RA, Georgieva RT. Earlier development of diabetic neuropathy in men than in women with type 2 diabetes mellitus. *Gend Med* 2010;7(6):600-15. doi: 10.1016/j.genm.2010.11.001

38. Stirling PHC, Jenkins PJ, Clement ND, et al. The Influence of Self-Reported Hand-Arm Vibration Exposure on Functional Outcomes Following Carpal Tunnel Release. *J Hand Surg Am* 2020;45(11):1029-36. doi: 10.1016/j.jhsa.2020.08.018

39. Stirling PHC, Jenkins PJ, McEachan JE. Previous vibration exposure in patients undergoing surgical treatment of Dupuytren's contracture. *J Hand Surg Eur Vol*

For peer review only

2020;45(5):525-27. doi: 10.1177/1753193420903675 [published Online First: 20200130]

40. Stirling PHC, Ng N, Jenkins PJ, et al. Hand-arm vibration and outcomes of surgery for Dupuytren's contracture. *Occup Med (Lond)* 2021;71(4-5):219-22. doi: 10.1093/occmed/kqab070

For peer review only

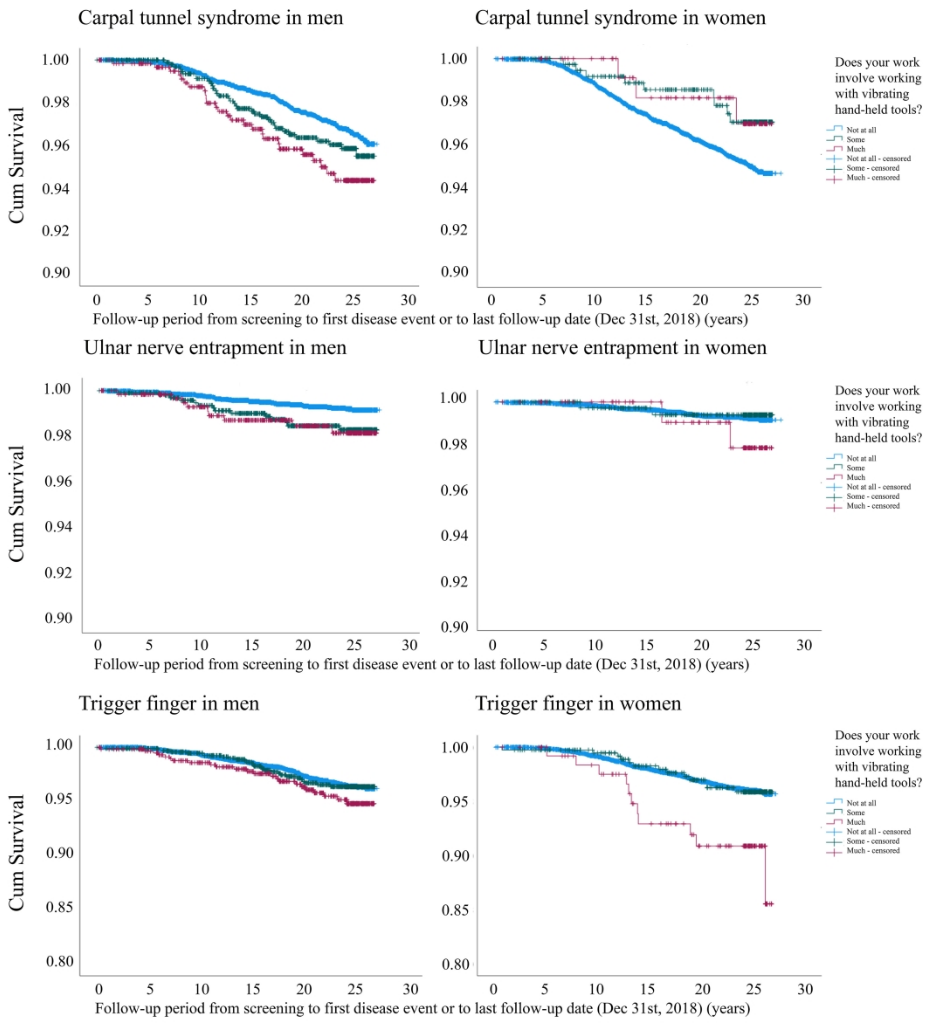

355x373mm (300 x 300 DPI)

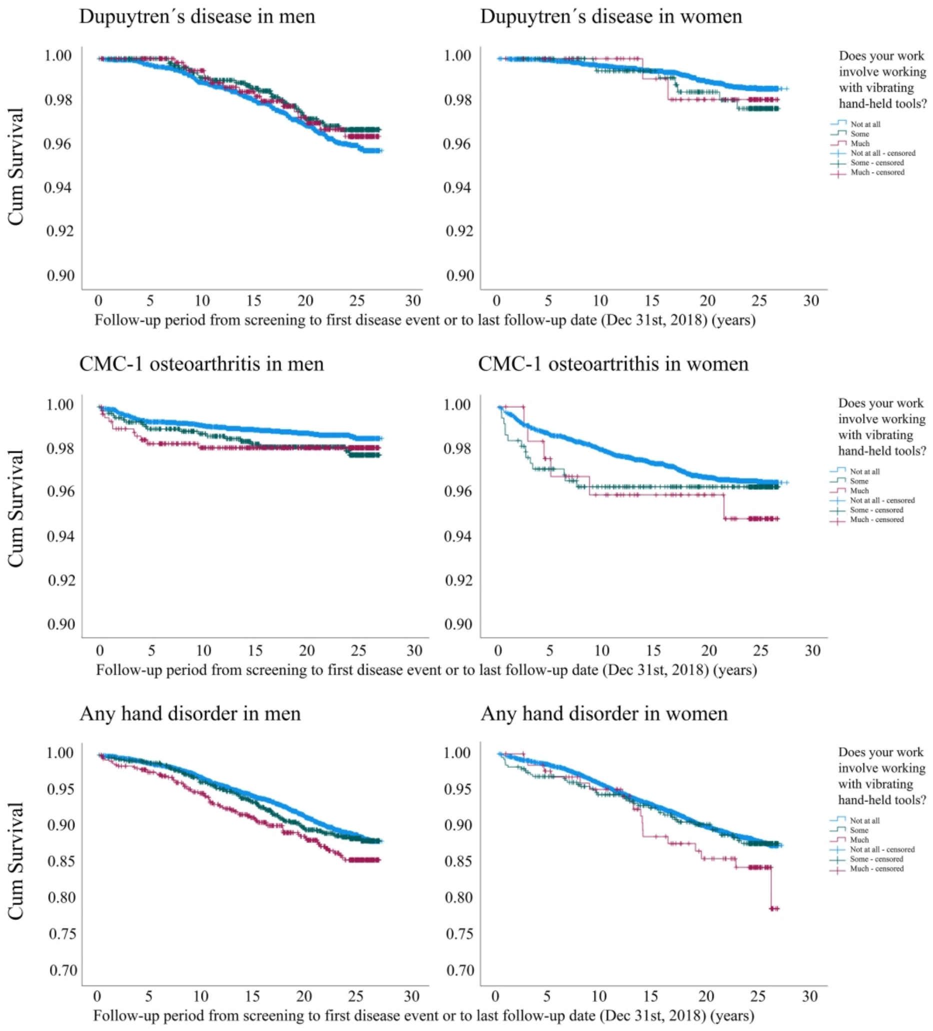

355x376mm (300 x 300 DPI)

STROBE 2007 (v4) checklist of items to be included in reports of observational studies in epidemiology*
Checklist for cohort, case-control, and cross-sectional studies (combined)

Section/Topic	Item #	Recommendation	Reported on page #
**Title and abstract**	1	(*a*) Indicate the study's design with a commonly used term in the title or the abstract	1-2
		(*b*) Provide in the abstract an informative and balanced summary of what was done and what was found	2-3
**Introduction**			
Background/rationale	2	Explain the scientific background and rationale for the investigation being reported	4-5
Objectives	3	State specific objectives, including any pre-specified hypotheses	5
**Methods**			
Study design	4	Present key elements of study design early in the paper	5
Setting	5	Describe the setting, locations, and relevant dates, including periods of recruitment, exposure, follow-up, and data collection	5
Participants	6	(*a*) *Cohort study*—Give the eligibility criteria, and the sources and methods of selection of participants. Describe methods of follow-up<br>*Case-control study*—Give the eligibility criteria, and the sources and methods of case ascertainment and control selection. Give the rationale for the choice of cases and controls<br>*Cross-sectional study*—Give the eligibility criteria, and the sources and methods of selection of participants	5-6
		(*b*) *Cohort study*—For matched studies, give matching criteria and number of exposed and unexposed<br>*Case-control study*—For matched studies, give matching criteria and the number of controls per case	5-6
Variables	7	Clearly define all outcomes, exposures, predictors, potential confounders, and effect modifiers. Give diagnostic criteria, if applicable	5-6
Data sources/ measurement	8*	For each variable of interest, give sources of data and details of methods of assessment (measurement). Describe comparability of assessment methods if there is more than one group	5-6
Bias	9	Describe any efforts to address potential sources of bias	19
Study size	10	Explain how the study size was arrived at	5
Quantitative variables	11	Explain how quantitative variables were handled in the analyses. If applicable, describe which groupings were chosen and why	5-6
Statistical methods	12	(*a*) Describe all statistical methods, including those used to control for confounding	6
		(*b*) Describe any methods used to examine subgroups and interactions	6
		(*c*) Explain how missing data were addressed	6
		(*d*) *Cohort study*—If applicable, explain how loss to follow-up was addressed<br>*Case-control study*—If applicable, explain how matching of cases and controls was addressed	6

For peer review only

		Cross-sectional study—If applicable, describe analytical methods taking account of sampling strategy	
		(*e*) Describe any sensitivity analyses	-
**Results**			
Participants	13*	(a) Report numbers of individuals at each stage of study—eg numbers potentially eligible, examined for eligibility, confirmed eligible, included in the study, completing follow-up, and analysed	7-15
		(b) Give reasons for non-participation at each stage	-
		(c) Consider use of a flow diagram	included
Descriptive data	14*	(a) Give characteristics of study participants (eg demographic, clinical, social) and information on exposures and potential confounders	7-15
		(b) Indicate number of participants with missing data for each variable of interest	7-15
		(c) *Cohort study*—Summarise follow-up time (eg, average and total amount)	
Outcome data	15*	*Cohort study*—Report numbers of outcome events or summary measures over time	
		Case-control study—Report numbers in each exposure category, or summary measures of exposure	7-15
		Cross-sectional study—Report numbers of outcome events or summary measures	
Main results	16	(*a*) Give unadjusted estimates and, if applicable, confounder-adjusted estimates and their precision (eg, 95% confidence interval). Make clear which confounders were adjusted for and why they were included	7-15
		(*b*) Report category boundaries when continuous variables were categorized	7-15
		(*c*) If relevant, consider translating estimates of relative risk into absolute risk for a meaningful time period	
Other analyses	17	Report other analyses done—eg analyses of subgroups and interactions, and sensitivity analyses	-
**Discussion**			
Key results	18	Summarise key results with reference to study objectives	15-16
Limitations	19	Discuss limitations of the study, taking into account sources of potential bias or imprecision. Discuss both direction and magnitude of any potential bias	19
Interpretation	20	Give a cautious overall interpretation of results considering objectives, limitations, multiplicity of analyses, results from similar studies, and other relevant evidence	15-18
Generalisability	21	Discuss the generalisability (external validity) of the study results	19
**Other information**			
Funding	22	Give the source of funding and the role of the funders for the present study and, if applicable, for the original study on which the present article is based	20

*Give information separately for cases and controls in case-control studies and, if applicable, for exposed and unexposed groups in cohort and cross-sectional studies.

Note: An Explanation and Elaboration article discusses each checklist item and gives methodological background and published examples of transparent reporting. The STROBE checklist is best used in conjunction with this article (freely available on the Web sites of PLoS Medicine at http://www.plosmedicine.org/, Annals of Internal Medicine at http://www.annals.org/, and Epidemiology at http://www.epidem.com/). Information on the STROBE Initiative is available at www.strobe-statement.org.

For peer review only

Risk of hand and forearm conditions due to vibrating hand-held tools exposure – a retrospective cohort study from Sweden

Journal:	*BMJ Open*
Manuscript ID	bmjopen-2023-080777.R1
Article Type:	Original research
Date Submitted by the Author:	10-Feb-2024
Complete List of Authors:	Zimmerman, Malin; Lund University, Department of Translational Medicine – Hand Surgery; Helsingborgs lasarett, Department of Orthopedics<br>Nilsson, Peter; Lund University, Clinical Sciences<br>Rydberg, Mattias; Lund University, Department of Translational Medicine; Skånes universitetssjukhus Malmö, Department of Hand Surgery<br>Dahlin, Lars; Lund University, Department of Translational Medicine; Skånes universitetssjukhus Malmö, Department of Hand Surgery
**Primary Subject Heading**:	Occupational and environmental medicine
Secondary Subject Heading:	Epidemiology, Neurology, Occupational and environmental medicine
Keywords:	Hand & wrist < ORTHOPAEDIC & TRAUMA SURGERY, Musculoskeletal disorders < ORTHOPAEDIC & TRAUMA SURGERY, General diabetes < DIABETES & ENDOCRINOLOGY, Hypertension < CARDIOLOGY, Aged

SCHOLARONE™
Manuscripts

I, the Submitting Author has the right to grant and does grant on behalf of all authors of the Work (as defined in the below author licence), an exclusive licence and/or a non-exclusive licence for contributions from authors who are: i) UK Crown employees; ii) where BMJ has agreed a CC-BY licence shall apply, and/or iii) in accordance with the terms applicable for US Federal Government officers or employees acting as part of their official duties; on a worldwide, perpetual, irrevocable, royalty-free basis to BMJ Publishing Group Ltd ("BMJ") its licensees and where the relevant Journal is co-owned by BMJ to the co-owners of the Journal, to publish the Work in this journal and any other BMJ products and to exploit all rights, as set out in our licence.

The Submitting Author accepts and understands that any supply made under these terms is made by BMJ to the Submitting Author unless you are acting as an employee on behalf of your employer or a postgraduate student of an affiliated institution which is paying any applicable article publishing charge ("APC") for Open Access articles. Where the Submitting Author wishes to make the Work available on an Open Access basis (and intends to pay the relevant APC), the terms of reuse of such Open Access shall be governed by a Creative Commons licence – details of these licences and which Creative Commons licence will apply to this Work are set out in our licence referred to above.

Other than as permitted in any relevant BMJ Author's Self Archiving Policies, I confirm this Work has not been accepted for publication elsewhere, is not being considered for publication elsewhere and does not duplicate material already published. I confirm all authors consent to publication of this Work and authorise the granting of this licence.

For peer review only

Risk of hand and forearm conditions due to vibrating hand-held tools exposure – a retrospective cohort study from Sweden

Malin Zimmerman, MD, PhD, Assoc. Prof. [1,2,3], Peter M. Nilsson, MD, PhD, Prof. [4], Mattias Rydberg, MD, PhD [1,3], Lars B. Dahlin, MD, PhD, Prof [1,3,5*]

[1] Department of Translational Medicine – Hand Surgery, Lund University, Malmö, Sweden

[2] Department of Orthopedics, Helsingborg's Hospital, Helsingborg, Sweden

[3] Department of Hand Surgery, Skåne University Hospital, Malmö, Sweden

[4] Department of Clinical Sciences Malmö, Lund University, Malmö, Sweden

[5] Department of Biomedical and Clinical Sciences, Linköping University, Linköping, Sweden

* Corresponding author:

Lars B. Dahlin, Department of Translational Medicine – Hand Surgery, Lund University, Jan Waldenströms gata 5, 205 02 Malmö, Sweden; Fax +46 40 928855; lars.dahlin@med.lu.se

Word count: 2986

Keywords: carpal tunnel syndrome – osteoarthritis (OA) – Dupuytren's contracture – trigger finger disorder – ulnar nerve compression syndromes – vibration

For peer review only - http://bmjopen.bmj.com/site/about/guidelines.xhtml

Abstract

Objectives: The occurrence of hand and forearm disorders related to vibration exposure, adjusted for relevant background factors, is scarcely reported. We analyzed the prevalence of such conditions in a large population cohort, stratified by sex, and associations with exposure to vibrating hand-held tools.

Design: This is a retrospective cohort study.

Setting: Individuals in the Malmö Diet and Cancer Study cohort (MDCS; inclusion 1991-1996; followed until 2018) were asked, *"Does your work involve working with vibrating hand-held tools?"* (response: "Not at all", "Some" and "Much"). Data was cross-linked with national registers to identify treatment for carpal tunnel syndrome (CTS), ulnar nerve entrapment (UNE), Dupuytren's disease, trigger finger, or CMC-1osteoarthritis (OA). Cox regression models, unadjusted and adjusted (age, sex, prevalent diabetes, smoking, hypertension, and alcohol consumption), were performed to analyze the effects of reported vibration exposure.

Participants: Individuals in the MDCS who had answered the questionnaire on vibration exposure (14,342 out of the originally 30,446 individuals in MDCS) were included in the study.

Results: In total, 12,220/14,342 individuals (76%) reported "no" exposure, 1,392/14,342 (9%) "some" and 730/14,342 (5%) "much" exposure to vibrating hand-held tools. In men, "much" exposure was independently associated with CTS [Hazard Ratio; HR 1.71 (95% CI 1.11-2.62)] and UNE [HR 2.42 (1.15-5.07)]. "Some" exposure was independently associated with UNE in men [HR 2.10 (1.12-3.95)]. "Much" exposure was independently associated with trigger finger in women [2.73 (1.49-4.99)]. We found no effect of vibration exposure on Dupuytren's disease or CMC-1 OA. "Much" vibration exposure predicted any hand and forearm diagnosis in men [HR 1.44 (1.08-1.80)] but not in women.

For peer review only - http://bmjopen.bmj.com/site/about/guidelines.xhtml

Conclusions: Vibration exposure by hand-held tools increases the risk of developing CTS and UNE and any common hand and forearm conditions in men, whereas women only risk trigger finger and CMC-1 OA. Adjustment for relevant confounders in vibration exposure is crucial.

Strengths and limitations of this study:

- Different hand and forearm conditions, with long-term follow-up, were analyzed in the same population-based cohort of men and women, with calculated hazard ratios (HR) adjusted for relevant confounders.
- Quantification of vibration exposure is not possible in larger cohorts, but the simple grading (i.e., “no”, “some”, and “much”) showed an increased risk for some hand and forearm conditions.
- A 41% response rate was obtained among the population-based cohort, with no information on vibration exposure in >10 % of the individuals.
- Diagnoses were based on ICD-codes from the National Board of Health and Welfare, including both surgically and not surgically treated cases, but with no individuals from primary care.

For peer review only

INTRODUCTION

Hand-arm vibration syndrome (HAVS) consists of symptoms and functional, social, emotional, and psychological disability from injuries to various tissue components of the upper extremity, such as muscle, nerve, connective tissue, and joints (1-4). Vibration exposure is reported to induce pathological conditions, including carpal tunnel syndrome (CTS) (5, 6), ulnar nerve entrapment at the wrist or the elbow (UNE) (7), Dupuytren's contracture (8), or osteoarthritis of the first carpometacarpal joint (CMC-1) (7), all of which may severely affect the patient's hand function and ability to work. The most well-known symptoms are related to the blood vessels, where vibration exposure provokes vibration-induced white fingers (VWF) (9, 10).

Muscle weakness is a clinically common symptom that is described by individuals exposed to hand-held vibrating tools, and structural changes in the muscles have been described both in experimental studies and in human muscle biopsies (11). In addition, nerve biopsies from the posterior interosseous nerve just proximal to the wrist from patients exposed to vibrating hand-held tools indicate that vibration exposure induces neuropathy with substantial axonal loss (12). Such a neuropathy may be the underlying reason why exposed patients experience CTS-like symptoms or have a propensity to develop CTS (3). An increased occurrence of osteoarthritis in joints of the hand related to vibration exposure has been suggested (13). A less highlighted condition, although questioned to be work-related (14), and linked to exposure to vibrating hand-held tools is Dupuytren's disease with finger contracture affecting the palmar fascia and bending of fingers (8). However, a genetic component (15) as well as a relation to diabetes and occupation (16) may be more common causes of Dupuytren´s disease. Vibration exposure may promote or speed up the development of Dupuytren's contracture in a genetically predisposed individual.

For peer review only - http://bmjopen.bmj.com/site/about/guidelines.xhtml

Most previous studies report on one of the hand and forearm conditions, and few have reported the occurrence of all conditions suggested to be related to vibration exposure. We aimed to analyze the presence of five common hand and forearm disorders in a large population-based cohort, where a proportion of the individuals reported that they were "not at all" exposed or exposed to vibrating hand-held tools to "some" or "much" extent.

METHODS

Study population

The population-based register from southern Sweden, the Malmö Diet and Cancer Study (MDCS), which has previously been described (17, 18), was used in this observational study. In brief, 30,446 individuals aged 46-73 were invited to participate between 1991-1996. Initially, individuals were screened for cardiovascular risk factors, undergoing clinical examination and laboratory assessment under fasting conditions. In addition, they responded to several questionnaires. One of the questionnaires contained the question, *"Does your work involve working with vibrating hand-held tools?"* where the individuals responded, *"Not at all"*, *"Some"* and *"Much"*. This questionnaire was answered by 14,342 individuals included in the present study. Hence, we report self-reported vibration exposure. In addition, other relevant variables were noted, including smoking, alcohol consumption, hypertension, and diabetes mellitus. Smoking was self-reported at baseline. Alcohol consumption was self-reported use during the last week at baseline and reported as g/day. Diabetes data was obtained through linkage with the National Diabetes Register (NDR), which contains data from both hospital care and primary health care on patients with diabetes. The present or latest job was defined according to the Swedish socioeconomic classification into three groups; employees (including officials/salaried employees), laborers (including laborers in the production of goods or services as well as farmers), or self-employed. Non-participants of the

For peer review only - http://bmjopen.bmj.com/site/about/guidelines.xhtml

MDCS are best described in the study by Manjer et al (18), where mortality was higher in non-participants, which might reflect a larger disease burden and less willingness to participate.

Endpoint retrieval

In the present study, we used data from national registers managed by the National Board of Health and Welfare to identify whether the included individuals had been treated for the hand and forearm conditions of interest. The used registers include data from hospitals and specialized care settings (outpatient clinics) but not from primary care units. Inpatient care has been registered since 1964, outpatient surgeries are included from 1997, and visits in specialized outpatient clinics are included from 2001. The included hand and forearm conditions were the two most common nerve compression disorders [carpal tunnel syndrome (ICD-10 code G56.0) and ulnar nerve entrapment at the elbow or the wrist (G56.2)], Dupuytren's disease with contracture (M72.0), trigger finger (M65.3) and osteoarthritis of the first CMC joint (M18.X). Patients were followed until death, emigration, or end of study 2018-12-31, whichever occurred first.

Statistical analysis

The baseline characteristics are presented as median (25th to 75th percentiles) or proportion of patients n (%). Since the continuous variables (age and alcohol consumption) were not normally-distributed, we used non-parametric testing. The Kruskal-Wallis test was used to detect any statistical significance between groups based on vibration exposure for continuous variables. If p-value <0.05, a Mann-Whitney U-test was used for pairwise individual comparisons between the three groups. For ordinal variables, the Chi^2-test was used for group comparisons. Cox regression analysis was performed to obtain Hazard Ratios (HR) for risk of

For peer review only - http://bmjopen.bmj.com/site/about/guidelines.xhtml

development of hand and forearm pathology due to vibration exposure. Time to event was defined as follow-up period from screening to first disease event or to last follow-up date (2018-12-31; years), emigration or death. In the group with "much" vibration exposure, there were 82% men, hence the results were stratified by sex. The group without vibration exposure was used as a reference group. We also analyzed vibration exposure as a dichotomous variable ("no" vibration exposure vs. "any" vibration exposure). The first models were unadjusted. The second models were adjusted for age at baseline, sex, prevalent diabetes before the study started, smoking, hypertension, and alcohol consumption. Confounders were chosen based on previous data and the authors' clinical experience on influencing factors for the specific diagnosis (19-21) (see also Discussion). Finally, Kaplan Meier curves were used to visualize disease-free survival. All statistical analysis was made using IBM SPSS Statistics for Mac version 28 (SPSS Inc., Chicago, IL, USA), and a two-sided p-value <0.05 was considered significant.

Patient and Public Involvement Statement

No patients, politicians, or the public were involved in elaborating on the present research question or the design of the study. The obtained results, after publication in the scientific journal, will be disseminated in different ways.

RESULTS

Characteristics

In total, 12,220/14,342 individuals (85%) reported that they did not have had "any" exposure to vibrating hand-held tools, while 1,392/14,342 (10%) reported that they had "some" and 730/14,342 (5%) had "much" exposure to vibrating hand-held tools. Age was median 57±6 years in all groups. There were more men in the vibration exposed groups ["not at all"

For peer review only

4,845/12,220 (40%) vs 1,003/1,392 (72%) in the "some" group and 601/730 (82%) in the "much" group]. Most included individuals (7181/14,342; 50%) were employees, 5,440/14,342 (38%) were laborers, and 1,684/14,342 (12%) were self-employed. Occupational data was missing in 37 individuals. Basic characteristics are presented in supplemental table 1. Some differences in basic characteristics were observed among men and women related to the extent of reported vibration exposure (Table 1).

Carpal tunnel syndrome and ulnar nerve compression at the elbow or wrist

Among the men, CTS and UNE were more common in the groups with more vibration exposure; however, not being statistically significant for CTS (**Table 1**). In the Cox regression analysis, "much" vibration exposure was independently associated with CTS [Hazard Ratio 1.71 (95% CI 1.11-2.62] and UNE [HR 2.42 (1.15-5.07); **Table 2**]. "Any" vibration exposure was independently associated with a greater risk of CTS [HR 1.41 (1.02-1.95); **Table 2**]. "Some" vibration exposure was independently associated with UNE [HR 2.10 (1.12-3.95); **Table 2**]. Among the women, CTS seemed more common in the group without vibration exposure, however, it was not statistically significant. The Cox regression models for women showed no significant effects of vibration exposure on the risk of developing CTS and UNE (for survival analysis using Kaplan Meier curves in men and women regarding CTS and UNE see **Figures 1A -1D)**.

For peer review only

Table 1. Baseline characteristics of individuals that reported exposure to vibrating hand-held tools as "not at all", "some" and "much" stratified by sex.

	"Does your work involve working with vibrating hand-held tools?"							
	**Men (n=6448)**				**Women (n=7892)**			
	**"Not at all" n=4,845**	**"Some" n=1,003**	**"Much" n=601**	**P-value**	**"Not at all" n=7,374**	**"Some" n=389**	**"Much" n=129**	**P-value**
Age (years)	57±6	57±6	57±6	0.67	57±6	58±6	57±6	0.079
Current smoking	1,355 (28%)	355 (35%)	211 (35%)	**<0.001†,‡**	2,103 (29%)	122 (31%)	46 (36%)	0.10
Antihypertensive treatment	2,499 (52%)	529 (53%)	329 (55%)	0.306	3,264 (44%)	179 (46%)	44 (34%)	0.053
Prevalent diabetes	269 (6%)	69 (7%)	33 (6%)	0.249	277 (4%)	16 (4%)	4 (3%)	0.87
Alcohol consumption (g/day)	12.2 [4.5-23.0]	9.6 [2.9-21.3]	10.6 [1.8-20.1]	**<0.001†,‡**	5.4 [0.8-11.2]	4.4 [0.46-10.0]	4.1 [0.0-10.3]	**0.019†**

For peer review only - http://bmjopen.bmj.com/site/about/guidelines.xhtml

Laborers (n, %)	1234 (25%)	630 (63%)	472 (79%)	**<0.001**†,‡	2764 (37%)	263 (68%)	77 (60%)	**<0.001**†,‡
Incident carpal tunnel syndrome (CTS)	133 (2.7%)	34 (3.4%)	26 (4.3%)	0.072	327 (4.4%)	9 (2.3%)	3 (2.3%)	0.071
Incident ulnar nerve entrapment at elbow/wrist (UNE)	33 (0.7%)	14 (1.4%)	9 (1.5%)	**0.019**‡	51 (0.7%)	2 (0.5%)	2 (1.6%)	0.46
Incident Dupuytren's contracture	158 (3.3)	25 (2.5%)	16 (2.7%)	0.36	87 (1.2)	7 (1.8%)	2 (1.6%)	0.52
Incident trigger finger	137 (2.8%)	28 (2.8%)	23 (3.8%)	0.38	262 (3.6%)	13 (3.3%)	11 (8.5%)	**0.011**‡,§
Incident osteoarthritis of the thumb CMC-1 joint	61 (1.3%)	19 (1.9%)	11 (1.8%)	0.20	236 (3.2%)	14 (3.6%)	6 (4.7%)	0.60
Any incident hand and forearm disorder	442 (9.1%)	94 (9.4%)	69 (11.5%)	0.17	793 (10.8%)	41 (10.5%)	18 (14%)	0.50

For peer review only - http://bmjopen.bmj.com/site/about/guidelines.xhtml

P-value for good comparisons between the subjects are based on Kruskal Wallis and Mann-Whitney U-test as a posthoc test and Chi2 test for ordinal variables. † Statistical significance was found between “not at all” and “some”. ‡ Statistical significance was found between “not at all” and “much”. § Statistical significance found between “some” and “much”.

For peer review only

Table 2. Cox regression models – unadjusted and adjusted for relevant factors – with the association between vibration exposure and risk for development of five specific hand and forearm conditions and any of the hand and forearm conditions stratified by sex.

**Men**						
Model 1 - unadjusted						
	**CTS**	**UNE**	**Trigger finger**	**Dupuytren's contracture**	**CMC-1 osteoarthritis**	**Any hand and forearm condition**
"No" vibration exposure	*Reference*					
"Any" vibration exposure	1.43 (1.04-1.97)	1.18 (0.69-2.04)	1.22 (0.88-1.70)	1.10 (0.78-1.57)	1.27 (0.82-1.97)	1.30 (1.08-1.55)
"Some" vibration exposure	1.21 (0.82-1.79)	2.20 (1.17-4.12)	1.01 (0.67-1.53)	0.78 (0.51-1.20)	1.57 (0.94-2.64)	1.05 (0.84-1.32)
"Much" vibration exposure	1.71 (1.11-2.62)	2.44 (1.17-5.12)	1.50 (0.97-2.34)	0.90 (0.54-1.50)	1.55 (0.82-2.95)	1.40 (1.08-1.80)
Model 2 – adjusted for age, smoking, alcohol consumption, hypertension and diabetes						
"No" vibration exposure	Reference					
"Any" vibration exposure	1.41 (1.02-1.95)	0.84 (0.44-1.60)	1.22 (0.88-1.70)	1.15 (0.81-1.65)	1.56 (0.97-2.50)	1.33 (1.10-1.60)
"Some" vibration exposure	1.18 (0.80-1.76)	2.10 (1.12-3.95)	1.01 (0.67-1.53)	0.82 (0.53-1.27)	1.55 (0.92-2.61)	1.06 (0.84-1.33)
"Much" vibration exposure	1.71 (1.11-2.62)	2.42 (1.15-5.07)	1.54 (0.99-2.39)	0.96 (0.57-1.61)	1.55 (0.81-2.94)	1.44 (1.12-1.86)

For peer review only - http://bmjopen.bmj.com/site/about/guidelines.xhtml

**Women**						
Model 1 - unadjusted						
	**CTS**	**UNE**	**Trigger finger**	**Dupuytren's contracture**	**CMC-1 osteoarthritis**	**Any hand and forearm condition**
"No" vibration exposure	*Reference*					
"Any" vibration exposure	0.85 (0.48-1.52)	0.89 (0.32-2.50)	1.08 (0.71-1.64)	0.85 (0.43-1.70)	2.06 (1.29-3.27)	1.12 (0.86-1.46)
"Some" vibration exposure	0.56 (0.29-1.08)	0.83 (0.20-3.40)	1.02 (0.59-1.78)	1.67 (0.77-3.60)	1.20 (0.70-2.06)	1.06 (0.77-1.45)
"Much" vibration exposure	0.56 (0.18-1.75)	2.50 (0.61-10.26)	2.72 (1.49-4.96)	1.43 (0.35-5.83)	1.55 (0.69-3.48)	1.43 (0.89-2.28)
Model 2 – adjusted for age, smoking, alcohol consumption, hypertension, and diabetes						
"No" vibration exposure	Reference					
"Any" vibration exposure	0.86 (0.48-1.54)	0.78 (0.25-2.44)	1.06 (0.70-1.63)	0.71 (0.34-1.51)	1.96 (1.23-3.12)	1.09 (0.83-1.42)
"Some" vibration exposure	0.56 (0.29-1.09)	0.82 (0.20-3.36)	1.05 (0.60-1.83)	1.76 (0.81-3.80)	1.19 (0.69-2.03)	1.07 (0.78-1.46)
"Much" vibration exposure	0.56 (0.18-1.76)	2.54 (0.62-10.48)	2.73 (1.49-4.99)	1.57 (0.39-6.38)	1.57 (0.70-3.54)	1.45 (0.91-2.32)

CTS; carpal tunnel syndrome, UNE; ulnar nerve entrapment at elbow/wrist, CMC-1; first carpometacarpal joint

For peer review only

Figure 1. Survival analysis using Kaplan Meier curves of the studied hand and forearm conditions that were followed from screening to the first disease event or to the last follow-up date (Dec 31st, 2018) and were potentially associated with vibration exposure stratified by sex. The individuals replied to the question "Does your work involve working with vibrating hand-held tools?" with possible answers "Not at all", "Some" and "Much".

Trigger finger

Trigger finger was more common among women with "much" vibration exposure than women with "some" or "no" vibration exposure (**Table 1**). No differences could be seen between the groups of men. "Much" vibration exposure was independently associated with the development of trigger finger in women [HR 2.72 (1.49-4.96)], but not in men (**Table 2;** for survival analysis using Kaplan Meier curves **see Figures 1E -1F**).

Dupuytren's contracture

Dupuytren's contracture was equally common among the groups in both sexes, and no significant effect of vibration exposure on the development of Dupuytren's disease with contracture could be demonstrated in the Cox regression analysis (**Table 1** and **2;** for survival analysis using Kaplan Meier curves **see Figures 1G - 1H**).

Osteoarthritis of the first CMC joint

CMC-1 OA was equally common among the groups in both sexes. In women, "any" vibration exposure was independently associated with the risk of CMC-1 OA [HR 1.96 (1.23-3.12)] in the Cox regression analysis (**Table 1** and **2;** for survival analysis using Kaplan Meier curves **see Figures 1I - 1J**).

For peer review only

For peer review only - http://bmjopen.bmj.com/site/about/guidelines.xhtml

Any hand and forearm condition

We found similar proportions of any incident hand and forearm condition among all groups (**Table 1**). In the Cox regression analysis, "much" vibration exposure predicted any hand and forearm condition in men [HR 1.44 (1.12-1.86)] but not in women [HR 1.45 (0.91-2.32)] (**Table 2;** for survival analysis using Kaplan Meier curves **see Figures 1K -1L**). "Any" vibration exposure was associated with a greater risk of any hand and forearm condition in men [HR 1.33(1.10-1.60) (**Table 2**).

DISCUSSION

The present observational study of a large population-based cohort, where the individuals replied to a question concerning exposure to vibrating hand-held tools and graded as "not at all", "some" and "much", shows that hand and forearm conditions in men, such as CTS and UNE, were significantly higher than in un-exposed men, particularly among those with "much" vibration exposure when adjusted for several relevant confounding factors. In addition, any hand and forearm condition was associated with "any" and "much" exposure to vibrating hand-held tools. In contrast, in women, there was an association between "any" exposure to vibrating hand-held tools and CMC-1 OA, and an association between "much" vibration exposure and trigger finger. The presently described hand and forearm conditions are common in the general population, irrespective of vibration exposure, where prevalence figures for CTS, UNE, Dupuytren´s contracture, trigger finger, and osteoarthritis of the CMC-1 joint are reported as 1.4-15 % (20, 22, 23). Previously published data has mainly included one of the present diagnoses at a time associated to vibration exposure, while the data from the current cohort also allowed the inclusion of all the relevant, and previously discussed,

For peer review only - http://bmjopen.bmj.com/site/about/guidelines.xhtml

For peer review only

conditions in the hand and forearm associated to exposure to vibrating hand-held tools (8, 24-26).

Risk assessment in vibration exposure of hand-held tools to different hand and forearm disorders may be complex due to the high prevalence of some of the hand and forearm disorders as well as the association with relevant factors and general comorbidities that must be adjusted for in any statistical analysis (8, 13, 21, 26, 27). In this study, we stratified our data on sex due to the relatively high number of male individuals in the cohort, as well attempting to level out any difference in occurrence of vibration exposure in men and women as sex is known to influence the prevalence of hand and forearm diagnoses (28). In our *Model 2*, we adjusted for factors that may be of relevance in development of the included hand and forearm disorders, i.e., age, smoking, alcohol consumption, hypertension, and diabetes; variables that are accessible in the MDCS database (19, 27). Age is a highly relevant influencing factor for development hand and forearm disorders, where men and women have different age distributions for development of CTS and UNE as well as of osteoarthritis in the first CMC joint (23, 28, 29). In contrast to our study, other authors have reported an increased risk for CTS in both sexes, although being highest among young men (24), which may be explained by differences between the cohorts and adjustment of confounding factors. Smoking may affect both the development and outcome of surgery of CTS and UNE (27), due to its effect on the intraneural microcirculation as well as being highlighted in Dupuytren´s disease with contracture (30). Individuals with hypertension may also have a higher perfusion pressure in the intraneural blood vessels making the nerve more resistant to compression (31); again, indicating that adjustment for several factors in the Cox analysis is necessary (32). Development of CTS, UNE, Dupuytren´s disease with finger contracture, and trigger fingers are also associated with both type 1 and type 2 diabetes (19), which is probably not entirely

For peer review only - http://bmjopen.bmj.com/site/about/guidelines.xhtml

true for osteoarthritis of the first CMC joint, for which high BMI and obesity are significant risk markers/factors (33). Finally, alcohol consumption is also a factor that may be involved in development of Dupuytren´s contracture as well as a cause of UNE (19, 34). Thus, it is crucial to adjust for age, smoking, alcohol consumption, hypertension, and diabetes when analyzing the risk of developing various hand and forearm disorders in relation to vibration exposure of hand-held tools. We also included the association of any hand and forearm disorder as a variable in relation to vibration exposure, which is appropriate since there usually is an extensive co-morbidity of various hand and forearm disorders, such as concomitant CTS in surgically treated patients with UNE, as well as the other present hand and forearm conditions (35). In addition, a subject with diabetes, irrespective of having type 1 or type 2 diabetes or if neuropathy is present, may have an increased susceptibility to a nerve compression disorders as a subject with a history of vibration exposure.

The observed differences in risks for developing the studied hand and forearm conditions in men and women are interesting and may have different explanations. Surprisingly, in women, "much" vibration exposure only increased the risk of trigger finger, while in men, the risks were higher for CTS and UNE. Women also differed in the marked risk for CMC-1 OA with "any" vibration exposure. There are no apparent reasons for this discrepancy. Still, it has been suggested that an underlying neuropathy may be important for development of CTS in vibration-exposed individuals (3, 8). One other study, however, found that working with vibrating tools for more than two hours per day was a risk factor for developing CTS in women but not in men (36). Furthermore, neuropathy is reported to be more common and more severe in men with diabetes (24, 37). Therefore, one may speculate that similar mechanisms may explain the present sex difference concerning nerve compression disorders. Trigger finger is more common among women, and it has been speculated that estrogen

For peer review only

deficiency alters tendon metabolism in postmenopausal women, hence impacting the risk of developing trigger finger (20). Previous studies have failed to demonstrate an association between occupational factors and trigger finger (20). It is, however, possible that during the early 1990s, when this cohort study started, occupational sex differences of risk existed, and among the participants who worked with vibrating hand-held tools, men worked with large tools, such as grinding machines and chainsaws. In contrast, women may have worked more with precision dental drills and other smaller tools. Whether the type of tool affects these disorders' development is unknown.

Our data also indicates that there is a greater risk of osteoarthritis in the first CMC joint in vibration-exposed women. In contrast, one older study investigated radiological osteoarthritis in vibration exposed and non-exposed individuals, and found no significant differences between groups, indicating that the development of such a condition has other causes than vibration exposure alone (25). It is, however, unclear whether that study included any women. One more recent meta-analysis could not demonstrate any increased risk of hand OA from vibration exposure (13). However, women have been underrepresented in many studies on vibration exposure. Also, it is possible that vibration exposure in women more often comes from precision instruments, such as dental instruments that require a pinch grip, as indicated by the higher proportion of laborers among men with “much” vibration exposure compared to the lower proportion of laborers among women with “much” vibration exposure. Tasks requiring much pinch strength have been associated with an increased risk of hand OA (13).

It was not possible and was not the present study’s purpose to evaluate any surgery outcome for the conditions in the individuals with “no”, “some”, or “much” exposure to vibration. The result of surgery in vibration-exposed patients with CTS seems to be impaired compared to

For peer review only - http://bmjopen.bmj.com/site/about/guidelines.xhtml

otherwise healthy patients with CTS (38). Still, outcome data are essentially lacking for the other hand and forearm conditions included in the present study, except for Dupuytren´s contracture, for which socioeconomic status has to be considered (39). Earlier exposure to vibrating tools may affect the pre- and postoperative function in individuals undergoing surgery for Dupuytren's contracture. However, the satisfaction or return to work was not different (40). Hence, whether vibration exposure affects clinical outcomes following treatment for common hand and forearm conditions remains an important area for future research.

Limitations

There are some limitations of the study, such as that in >10 % of the individuals, no information was available concerning vibration exposure as well as a 41% response rate among the population-based cohort (18). This might limit the generalizability of this study and there might exist a selection bias in who answered the questionnaires. In addition, our diagnoses were based on ICD-codes from the National Board of Health and Welfare, where both surgically and not surgically treated cases were included, although without any patients diagnosed in primary care. This might include a selection bias, since patients with milder forms of the studied hand and forearm conditions that can be treated conservatively are managed in primary care. Quantifying the vibration exposure is relevant but is not possible in larger cohorts as in the present study, which is a limitation. We also had no data on hand dominance, nor ethnicity and socioeconomic depravation that might influence prevalence of the studied conditions.

CONCLUSIONS

For peer review only - http://bmjopen.bmj.com/site/about/guidelines.xhtml

We conclude that vibration exposure to hand-held tools increases the risk of having or developing CTS, and particularly UNE, but also any common hand and forearm conditions in men and CMC-1 OA and trigger finger in women. It is essential to adjust for relevant influencing factors, such as age, smoking, alcohol consumption, hypertension, and diabetes, when investigating the risks of working with hand-held vibrating tools.

STATEMENTS

Author contributions

M.Z., P.N., and L.B.D. conceived the ideas; M.Z. and P.N. collected the data; M.Z., M.R., and L.B.D. analyzed the data; M.Z. and L.B.D. led the writing, and all authors contributed to the interpretation and the writing and approved the final version of the manuscript.

Funding statement

This research was funded by the Swedish Research Council (#2022-01942, principal investigator LBD), the Swedish Diabetes Foundation (#DIA2020-492, principal investigator LBD), the Regional Agreement on Medical Training and Clinical Research (ALF; PI LBD) between Region Skåne and Lund University (#2018-Projekt 0104; PI LBD) and Funds from Skåne University Hospital (#2019-659; PI LBD), Elly Olsson´s Foundation for scientific research (#N/A), Stig and Ragna Gorthon Foundation (#N/A), Almroth Foundation (#N/A), Kockska foundation (#N/A), and the Magnus Bergvall Foundation [2020-03612, principal investigator MZ].

The initial financial support to the Malmö Diet and Cancer Study cohort at baseline was provided by the Swedish Cancer Foundation (#N/A), The Swedish Medical Research Council (#N/A), The European Commission (#N/A), the City of Malmö (#N/A), the Swedish Dairy Association (#N/A) and the Albert Påhlsson Foundation (#N/A).

For peer review only - http://bmjopen.bmj.com/site/about/guidelines.xhtml

The sponsors had no role in the study design, collection, analysis, or interpretation of the data, writing the report, or decision to submit the manuscript.

Competing of Interests

The authors declare no conflict of interest.

Ethics Approval

Participants provided written informed consent before inclusion in MDCS. MDCS and the present study have been approved by the regional Ethics committee in Lund and the Swedish Ethical Review Authority (DNR: LU51-90; 2009-633; 2019-01439). The study was conducted in accordance with the Helsinki Declaration.

Data Sharing

The data are not publicly available due to ethical restrictions. The data that support the findings of this study are only available on request from the corresponding author as well as after application and approval by the Swedish Ethical Review Authority (https://etikprovningsmyndigheten.se/en/ due to the restrictions in Sweden, based on the national Law for ethical review of research on humans ["Lag (2003:460) om etikprövning av forskning som avser människor"].

Acknowledgments

The authors are very grateful to all the participants in MDCS whose participation enabled this work. We are also thankful to the National Diabetes Register (NDR) and Statistics Sweden (SCB) for register data, Anders Dahlin for his help with data extraction, and Tina Folker for her administrative assistance. We acknowledge support from the Lund University Infrastructure grant "Malmö population-based cohorts" (STYR 2019/2046).

For peer review only

References

1. Dong RG, Rakheja S, Schopper AW, Han B, Smutz WP. Hand-transmitted vibration and biodynamic response of the human hand-arm: a critical review. Crit Rev Biomed Eng. 2001;29(4):393-439.
2. Pelmear P, Leong D. Review of occupational standards and guidelines for hand-arm (segmental) vibration syndrome (HAVS). Appl Occup Environ Hyg. 2000;15(3):291-302.
3. Dahlin LB, Sanden H, Dahlin E, Zimmerman M, Thomsen N, Bjorkman A. Low myelinated nerve-fibre density may lead to symptoms associated with nerve entrapment in vibration-induced neuropathy. Journal of occupational medicine and toxicology (London, England). 2014;9(1):7.
4. Lundborg G, Dahlin LB, Hansson H-A, Kanje M, Necking LE. Vibration exposure and peripheral nerve fiber damage. J Hand Surg. 1990;15A:346-51.
5. Hulkkonen S, Shiri R, Auvinen J, Miettunen J, Karppinen J, Ryhanen J. Risk factors of hospitalization for carpal tunnel syndrome among the general working population. Scand J Work Environ Health. 2020;46(1):43-9.
6. Lawson IJ. Is carpal tunnel syndrome caused by work with vibrating tools? Occup Med (Lond). 2020;70(1):8-10.
7. Piligian G, Herbert R, Hearns M, Dropkin J, Landsbergis P, Cherniack M. Evaluation and management of chronic work-related musculoskeletal disorders of the distal upper extremity. Am J Ind Med. 2000;37(1):75-93.
8. Mathieu S, Naughton G, Descatha A, Soubrier M, Dutheil F. Dupuytren's Disease and exposure to vibration: Systematic review and Meta-analysis. Joint Bone Spine. 2020;87(3):203-7.
9. Aarhus L, Stranden E, Nordby KC, Einarsdottir E, Olsen R, Ruud B, et al. Vascular component of hand-arm vibration syndrome: a 22-year follow-up study. Occup Med (Lond). 2018;68(6):384-90.
10. Wei N, Yan R, Lang L, Wei Y, Li J, Yang H, et al. Local vibration induced vascular pathological structural changes and abnormal levels of vascular damage indicators. Microvasc Res. 2021;136:104163.
11. Necking LE, Lundborg G, Lundström R, Thornell LE, Fridén J. Hand muscle pathology after long-term vibration exposure. J Hand Surg Br. 2004;29(5):431-7.
12. Stromberg T, Dahlin LB, Brun A, Lundborg G. Structural nerve changes at wrist level in workers exposed to vibration. Occup Environ Med. 1997;54(5):307-11.
13. Hammer PE, Shiri R, Kryger AI, Kirkeskov L, Bonde JP. Associations of work activities requiring pinch or hand grip or exposure to hand-arm vibration with finger and wrist osteoarthritis: a meta-analysis. Scandinavian journal of work, environment & health. 2014;40(2):133-45.
14. Descatha A, Jauffret P, Chastang JF, Roquelaure Y, Leclerc A. Should we consider Dupuytren's contracture as work-related? A review and meta-analysis of an old debate. BMC Musculoskelet Disord. 2011;12:96.

For peer review only - http://bmjopen.bmj.com/site/about/guidelines.xhtml

15. Ng M, Thakkar D, Southam L, Werker P, Ophoff R, Becker K, et al. A Genome-wide Association Study of Dupuytren Disease Reveals 17 Additional Variants Implicated in Fibrosis. Am J Hum Genet. 2017;101(3):417-27.
16. van den Berge BA, Wiberg A, Werker PMN, Broekstra DC, Furniss D. Dupuytren's disease is a work-related disorder: results of a population-based cohort study. Occup Environ Med. 2023;80(3):137-45.
17. Berglund G, Elmstahl S, Janzon L, Larsson SA. The Malmo Diet and Cancer Study. Design and feasibility. J Intern Med. 1993;233(1):45-51.
18. Manjer J, Carlsson S, Elmståhl S, Gullberg B, Janzon L, Lindström M, et al. The Malmö Diet and Cancer Study: representativity, cancer incidence and mortality in participants and non-participants. European journal of cancer prevention : the official journal of the European Cancer Prevention Organisation (ECP). 2001;10(6):489-99.
19. Rydberg M, Zimmerman M, Löfgren JP, Gottsäter A, Nilsson PM, Melander O, et al. Metabolic factors and the risk of Dupuytren's disease: data from 30,000 individuals followed for over 20 years. Sci Rep. 2021;11(1):14669.
20. Okita G, Hayashi M, Ikegami S, Iwakawa H, Haro H, Kato H. The Prevalence and Risk Factors for Trigger Digits in a Random Sampling of a Japanese Population Registry between 50 and 89 Years of Age. J Hand Surg Asian Pac Vol. 2022;27(1):148-55.
21. Jones G, Cooley HM, Stankovich JM. A cross sectional study of the association between sex, smoking, and other lifestyle factors and osteoarthritis of the hand. J Rheumatol. 2002;29(8):1719-24.
22. Haara MM, Heliovaara M, Kroger H, Arokoski JP, Manninen P, Karkkainen A, et al. Osteoarthritis in the carpometacarpal joint of the thumb. Prevalence and associations with disability and mortality. J Bone Joint Surg Am. 2004;86(7):1452-7.
23. Atroshi I, Gummesson C, Johnsson R, Ornstein E, Ranstam J, Rosen I. Prevalence of carpal tunnel syndrome in a general population [see comments]. JAMA. 1999;282(2):153-8.
24. Vihlborg P, Pettersson H, Makdoumi K, Wikstrom S, Bryngelsson IL, Selander J, et al. Carpal Tunnel Syndrome and Hand-Arm Vibration: A Swedish National Registry Case-Control Study. J Occup Environ Med. 2022;64(3):197-201.
25. Malchaire J, Maldague B, Huberlant JM, Croquet F. Bone and joint changes in the wrists and elbows and their association with hand and arm vibration exposure. Ann Occup Hyg. 1986;30(4):461-8.
26. van Rijn RM, Huisstede BM, Koes BW, Burdorf A. Associations between work-related factors and the carpal tunnel syndrome--a systematic review. Scandinavian journal of work, environment & health. 2009;35(1):19-36.
27. Pourmemari MH, Viikari-Juntura E, Shiri R. Smoking and carpal tunnel syndrome: a meta-analysis. Muscle Nerve. 2014;49(3):345-50.
28. Rydberg M, Zimmerman M, Gottsater A, Svensson AM, Eeg-Olofsson K, Dahlin LB. Diabetic hand: prevalence and incidence of diabetic hand problems using data from 1.1 million inhabitants in southern Sweden. BMJ Open Diabetes Res Care. 2022;10(1).
29. Mitake T, Iwatsuki K, Hirata H. Differences in characteristics of carpal tunnel syndrome between male and female patients. J Orthop Sci. 2020;25(5):843-6.
30. Eckerdal D, Nivestam A, Dahlin LB. Surgical treatment of Dupuytren's disease - outcome and health economy in relation to smoking and diabetes. BMC Musculoskelet Disord. 2014;15:117.
31. Szabo RM, Gelberman RH, Williamson RV, Hargens AR. Effects of increased systemic blood pressure on the tissue fluid pressure threshold of peripheral nerve. J Orthop Res. 1983;1(2):172-8.

For peer review only - http://bmjopen.bmj.com/site/about/guidelines.xhtml

32. Mustafa KN, Khader YS, Bsoul AK, Ajlouni K. Musculoskeletal disorders of the hand in type 2 diabetes mellitus: prevalence and its associated factors. International journal of rheumatic diseases. 2016;19(7):730-5.
33. Rydberg M, Dahlin LB, Gottsater A, Nilsson PM, Melander O, Zimmerman M. High body mass index is associated with increased risk for osteoarthritis of the first carpometacarpal joint during more than 30 years of follow-up. RMD Open. 2020;6(3).
34. Nordenskjold J, Englund M, Zhou C, Atroshi I. Prevalence and incidence of doctor-diagnosed Dupuytren's disease: a population-based study. J Hand Surg Eur Vol. 2017;42(7):673-7.
35. Giostad A, Nyman E. Patient Characteristics in Ulnar Nerve Compression at the Elbow at a Tertiary Referral Hospital and Predictive Factors for Outcomes of Simple Decompression versus Subcutaneous Transposition of the Ulnar Nerve. Biomed Res Int. 2019;2019:5302462.
36. Melchior M, Roquelaure Y, Evanoff B, Chastang JF, Ha C, Imbernon E, et al. Why are manual workers at high risk of upper limb disorders? The role of physical work factors in a random sample of workers in France (the Pays de la Loire study). Occup Environ Med. 2006;63(11):754-61.
37. Kamenov ZA, Parapunova RA, Georgieva RT. Earlier development of diabetic neuropathy in men than in women with type 2 diabetes mellitus. Gend Med. 2010;7(6):600-15.
38. Stirling PHC, Jenkins PJ, Clement ND, Duckworth AD, McEachan JE. The Influence of Self-Reported Hand-Arm Vibration Exposure on Functional Outcomes Following Carpal Tunnel Release. J Hand Surg Am. 2020;45(11):1029-36.
39. Stirling PHC, Jenkins PJ, McEachan JE. Previous vibration exposure in patients undergoing surgical treatment of Dupuytren's contracture. J Hand Surg Eur Vol. 2020;45(5):525-7.
40. Stirling PHC, Ng N, Jenkins PJ, Clement ND, Duckworth AD, McEachan JE. Hand-arm vibration and outcomes of surgery for Dupuytren's contracture. Occup Med (Lond). 2021;71(4-5):219-22.

For peer review only

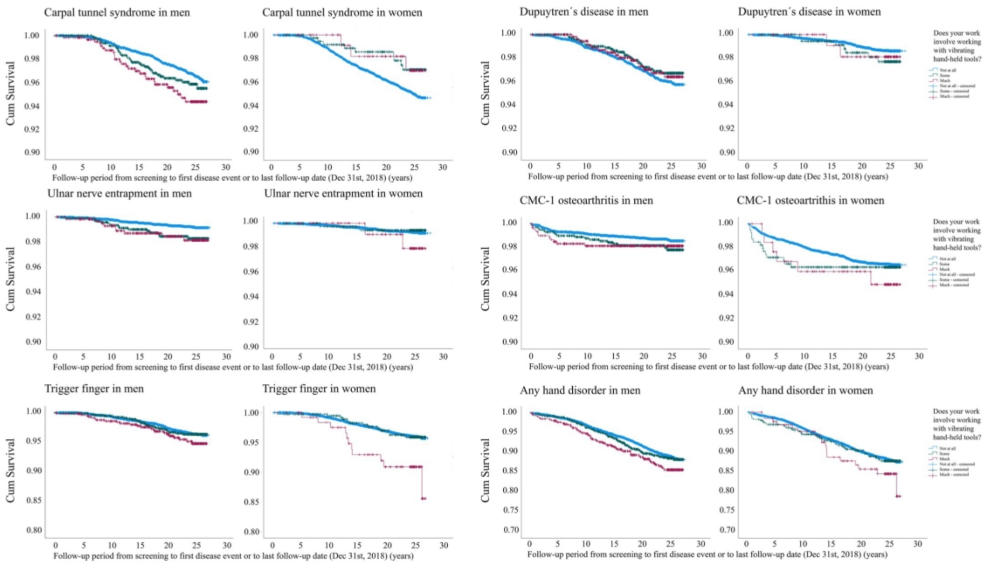

440x254mm (300 x 300 DPI)

Supplemental table 1. Baseline characteristics of all included individuals that reported exposure to vibrating hand-held tools as "not at all", "some" and "much".

	"Does your work involve working with vibrating hand-held tools?"			
	**"Not at all"**	**"Some"**	**"Much"**	**P-value**
	**12,220 (85%)**	**1,392 (10%)**	**730 (5%)**	
Age (years)	57±6	57±6	57±6	0.18
Sex (men)	4845 (40%)	1003 (72%)	601 (82%)	**<0.001**†,‡,§
Current smoking	3458 (28%)	477 (34%)	257 (35%)	**<0.001**†,‡
Antihypertensive treatment	5763 (47%)	708 (51%)	373 (51%)	**0.005**†,‡
Prevalent diabetes	546 (5%)	85 (6%)	37 (5%)	**0.02**†
Alcohol consumption (g/day)	7.3 [1.7-15.5]	8.1 [1.8-17.3]	9.0 [1.4-18.6]	**0.025**‡
Incident carpal tunnel syndrome (CTS)	460 (3.8%)	43 (3.1%)	29 (4.0%)	0.42
Incident ulnar nerve entrapment at elbow/wrist (UNE)	84 (0.7%)	16 (1.1%)	11 (1.5%)	**0.012**‡
Incident Dupuytren's contracture	245 (2.0)	32 (2.3%)	18 (2.5%)	0.56

For peer review only

Incident trigger finger	399 (3.3%)	41 (2.9%)	34 (4.7%)	0.091
Incident osteoarthritis of the thumb CMC-1 joint	297 (2.4%)	33 (2.4%)	17 (2.3%)	0.98
Any incident hand disorder	1,235 (10.1%)	135 (9.7%)	87 (11.9%)	0.24

P-value for good comparisons between the subjects are based on Kruskal Wallis and Mann-Whitney U-test as a posthoc test and Chi2 test for ordinal variables. † Statistical significance was found between "not at all" and "some". ‡ Statistical significance was found between "not at all" and "much". § Statistical significance found between "some" and "much".

For peer review only

STROBE 2007 (v4) checklist of items to be included in reports of observational studies in epidemiology*
Checklist for cohort, case-control, and cross-sectional studies (combined)

Section/Topic	Item #	Recommendation	Reported on page #
**Title and abstract**	1	(*a*) Indicate the study's design with a commonly used term in the title or the abstract	1-2
		(*b*) Provide in the abstract an informative and balanced summary of what was done and what was found	2-3
**Introduction**			
Background/rationale	2	Explain the scientific background and rationale for the investigation being reported	4-5
Objectives	3	State specific objectives, including any pre-specified hypotheses	5
**Methods**			
Study design	4	Present key elements of study design early in the paper	5
Setting	5	Describe the setting, locations, and relevant dates, including periods of recruitment, exposure, follow-up, and data collection	5
Participants	6	(*a*) *Cohort study*—Give the eligibility criteria, and the sources and methods of selection of participants. Describe methods of follow-up<br>*Case-control study*—Give the eligibility criteria, and the sources and methods of case ascertainment and control selection. Give the rationale for the choice of cases and controls<br>*Cross-sectional study*—Give the eligibility criteria, and the sources and methods of selection of participants	5-6
		(*b*) *Cohort study*—For matched studies, give matching criteria and number of exposed and unexposed<br>*Case-control study*—For matched studies, give matching criteria and the number of controls per case	5-6
Variables	7	Clearly define all outcomes, exposures, predictors, potential confounders, and effect modifiers. Give diagnostic criteria, if applicable	5-6
Data sources/ measurement	8*	For each variable of interest, give sources of data and details of methods of assessment (measurement). Describe comparability of assessment methods if there is more than one group	5-6
Bias	9	Describe any efforts to address potential sources of bias	19
Study size	10	Explain how the study size was arrived at	5
Quantitative variables	11	Explain how quantitative variables were handled in the analyses. If applicable, describe which groupings were chosen and why	5-6
Statistical methods	12	(*a*) Describe all statistical methods, including those used to control for confounding	6
		(*b*) Describe any methods used to examine subgroups and interactions	6
		(*c*) Explain how missing data were addressed	6
		(*d*) *Cohort study*—If applicable, explain how loss to follow-up was addressed<br>*Case-control study*—If applicable, explain how matching of cases and controls was addressed	6

For peer review only

		Cross-sectional study—If applicable, describe analytical methods taking account of sampling strategy	
		(*e*) Describe any sensitivity analyses	-
**Results**			
Participants	13*	(a) Report numbers of individuals at each stage of study—eg numbers potentially eligible, examined for eligibility, confirmed eligible, included in the study, completing follow-up, and analysed	7-15
		(b) Give reasons for non-participation at each stage	-
		(c) Consider use of a flow diagram	included
Descriptive data	14*	(a) Give characteristics of study participants (eg demographic, clinical, social) and information on exposures and potential confounders	7-15
		(b) Indicate number of participants with missing data for each variable of interest	7-15
		(c) *Cohort study*—Summarise follow-up time (eg, average and total amount)	
Outcome data	15*	*Cohort study*—Report numbers of outcome events or summary measures over time	
		Case-control study—Report numbers in each exposure category, or summary measures of exposure	7-15
		Cross-sectional study—Report numbers of outcome events or summary measures	
Main results	16	(*a*) Give unadjusted estimates and, if applicable, confounder-adjusted estimates and their precision (eg, 95% confidence interval). Make clear which confounders were adjusted for and why they were included	7-15
		(*b*) Report category boundaries when continuous variables were categorized	7-15
		(*c*) If relevant, consider translating estimates of relative risk into absolute risk for a meaningful time period	
Other analyses	17	Report other analyses done—eg analyses of subgroups and interactions, and sensitivity analyses	-
**Discussion**			
Key results	18	Summarise key results with reference to study objectives	15-16
Limitations	19	Discuss limitations of the study, taking into account sources of potential bias or imprecision. Discuss both direction and magnitude of any potential bias	19
Interpretation	20	Give a cautious overall interpretation of results considering objectives, limitations, multiplicity of analyses, results from similar studies, and other relevant evidence	15-18
Generalisability	21	Discuss the generalisability (external validity) of the study results	19
**Other information**			
Funding	22	Give the source of funding and the role of the funders for the present study and, if applicable, for the original study on which the present article is based	20

*Give information separately for cases and controls in case-control studies and, if applicable, for exposed and unexposed groups in cohort and cross-sectional studies.
Note: An Explanation and Elaboration article discusses each checklist item and gives methodological background and published examples of transparent reporting. The STROBE checklist is best used in conjunction with this article (freely available on the Web sites of PLoS Medicine at http://www.plosmedicine.org/, Annals of Internal Medicine at http://www.annals.org/, and Epidemiology at http://www.epidem.com/). Information on the STROBE Initiative is available at www.strobe-statement.org.

For peer review only

Risk of hand and forearm conditions due to vibrating hand-held tools exposure – a retrospective cohort study from Sweden

Journal:	*BMJ Open*
Manuscript ID	bmjopen-2023-080777.R2
Article Type:	Original research
Date Submitted by the Author:	06-May-2024

Complete List of Authors:	Zimmerman, Malin; Lund University, Department of Translational Medicine – Hand Surgery; Helsingborgs lasarett, Department of Orthopedics<br>Nilsson, Peter; Lund University, Clinical Sciences<br>Rydberg, Mattias; Lund University, Department of Translational Medicine; Skånes universitetssjukhus Malmö, Department of Hand Surgery<br>Dahlin, Lars; Lund University, Department of Translational Medicine; Skånes universitetssjukhus Malmö, Department of Hand Surgery

**Primary Subject Heading**:	Occupational and environmental medicine
Secondary Subject Heading:	Epidemiology, Neurology, Occupational and environmental medicine
Keywords:	Hand & wrist < ORTHOPAEDIC & TRAUMA SURGERY, Musculoskeletal disorders < ORTHOPAEDIC & TRAUMA SURGERY, General diabetes < DIABETES & ENDOCRINOLOGY, Hypertension < CARDIOLOGY, Aged

I, the Submitting Author has the right to grant and does grant on behalf of all authors of the Work (as defined in the below author licence), an exclusive licence and/or a non-exclusive licence for contributions from authors who are: i) UK Crown employees; ii) where BMJ has agreed a CC-BY licence shall apply, and/or iii) in accordance with the terms applicable for US Federal Government officers or employees acting as part of their official duties; on a worldwide, perpetual, irrevocable, royalty-free basis to BMJ Publishing Group Ltd ("BMJ") its licensees and where the relevant Journal is co-owned by BMJ to the co-owners of the Journal, to publish the Work in this journal and any other BMJ products and to exploit all rights, as set out in our licence.

The Submitting Author accepts and understands that any supply made under these terms is made by BMJ to the Submitting Author unless you are acting as an employee on behalf of your employer or a postgraduate student of an affiliated institution which is paying any applicable article publishing charge ("APC") for Open Access articles. Where the Submitting Author wishes to make the Work available on an Open Access basis (and intends to pay the relevant APC), the terms of reuse of such Open Access shall be governed by a Creative Commons licence – details of these licences and which Creative Commons licence will apply to this Work are set out in our licence referred to above.

Other than as permitted in any relevant BMJ Author's Self Archiving Policies, I confirm this Work has not been accepted for publication elsewhere, is not being considered for publication elsewhere and does not duplicate material already published. I confirm all authors consent to publication of this Work and authorise the granting of this licence.

For peer review only

Risk of hand and forearm conditions due to vibrating hand-held tools exposure – a retrospective cohort study from Sweden

Malin Zimmerman, MD, PhD, Assoc. Prof. [1,2,3], Peter M. Nilsson, MD, PhD, Prof. [4], Mattias Rydberg, MD, PhD [1,3], Lars B. Dahlin, MD, PhD, Prof [1,3,5*]

[1] Department of Translational Medicine – Hand Surgery, Lund University, Malmö, Sweden

[2] Department of Orthopedics, Helsingborg's Hospital, Helsingborg, Sweden

[3] Department of Hand Surgery, Skåne University Hospital, Malmö, Sweden

[4] Department of Clinical Sciences Malmö, Lund University, Malmö, Sweden

[5] Department of Biomedical and Clinical Sciences, Linköping University, Linköping, Sweden

* Corresponding author:

Lars B. Dahlin, Department of Translational Medicine – Hand Surgery, Lund University, Jan Waldenströms gata 5, 205 02 Malmö, Sweden; Fax +46 40 928855; lars.dahlin@med.lu.se

Word count: 3184

Keywords: carpal tunnel syndrome – osteoarthritis (OA) – Dupuytren's contracture – trigger finger disorder – ulnar nerve compression syndromes – vibration

For peer review only

Abstract

Objectives: The occurrence of hand and forearm disorders related to vibration exposure, adjusted for relevant background factors, is scarcely reported. We analyzed the prevalence of such conditions in a large population cohort, stratified by sex, and associations with exposure to vibrating hand-held tools.

Design: This is a retrospective cohort study.

Setting: Individuals in the Malmö Diet and Cancer Study cohort (MDCS; inclusion 1991-1996; followed until 2018) were asked, *"Does your work involve working with vibrating hand-held tools?"* (response: "Not at all", "Some" and "Much"). Data was cross-linked with national registers to identify treatment for carpal tunnel syndrome (CTS), ulnar nerve entrapment (UNE), Dupuytren's disease, trigger finger, or CMC-1osteoarthritis (OA). Cox regression models, unadjusted and adjusted (age, sex, prevalent diabetes, smoking, hypertension, and alcohol consumption), were performed to analyze the effects of reported vibration exposure.

Participants: Individuals in the MDCS who had answered the questionnaire on vibration exposure (14,342 out of the originally 30,446 individuals in MDCS) were included in the study.

Results: In total, 12,220/14,342 individuals (76%) reported "no" exposure, 1,392/14,342 (9%) "some" and 730/14,342 (5%) "much" exposure to vibrating hand-held tools. In men, "much" exposure was independently associated with CTS [Hazard Ratio; HR 1.71 (95% CI 1.11-2.62)] and UNE [HR 2.42 (1.15-5.07)]. "Some" exposure was independently associated with UNE in men [HR 2.10 (1.12-3.95)]. "Much" exposure was independently associated with trigger finger in women [2.73 (1.49-4.99)]. We found no effect of vibration exposure on Dupuytren's disease or CMC-1 OA. "Much" vibration exposure predicted any hand and forearm diagnosis in men [HR 1.44 (1.08-1.80)] but not in women.

For peer review only

Conclusions: Vibration exposure by hand-held tools increases the risk of developing CTS and UNE and any common hand and forearm conditions in men, whereas women only risk trigger finger and CMC-1 OA. Adjustment for relevant confounders in vibration exposure is crucial.

Strengths and limitations of this study:

- Different hand and forearm conditions, with long-term follow-up, were analyzed in the same population-based cohort of men and women, with calculated hazard ratios (HR) adjusted for relevant confounders.
- Quantification of vibration exposure is not possible in larger cohorts, but the simple grading (i.e., "no", "some", and "much") showed an increased risk for some hand and forearm conditions.
- A 41% response rate, which is a limitation, was obtained among the population-based cohort, with no information on vibration exposure in >10 % of the individuals.
- Diagnoses were based on ICD-codes from the National Board of Health and Welfare, including both surgically and not surgically treated cases, but with no individuals from primary care.

For peer review only - http://bmjopen.bmj.com/site/about/guidelines.xhtml

INTRODUCTION

Hand-arm vibration syndrome (HAVS) consists of symptoms and functional, social, emotional, and psychological disability from injuries to various tissue components of the upper extremity, such as muscle, nerve, connective tissue, and joints [1-4]. Vibration exposure is reported to induce pathological conditions, including carpal tunnel syndrome (CTS) [5, 6], ulnar nerve entrapment at the wrist or the elbow (UNE) [7], Dupuytren's contracture [8], or osteoarthritis of the first carpometacarpal joint (CMC-1) [7], all of which may severely affect the patient's hand function and ability to work. The most well-known symptoms are related to the blood vessels, where vibration exposure provokes vibration-induced white fingers (VWF) [9, 10].

Muscle weakness is a clinically common symptom that is described by individuals exposed to hand-held vibrating tools, and structural changes in the muscles have been described both in experimental studies and in human muscle biopsies [11]. In addition, nerve biopsies from the posterior interosseous nerve just proximal to the wrist from patients exposed to vibrating hand-held tools indicate that vibration exposure induces neuropathy with substantial axonal loss [12]. Such a neuropathy may be the underlying reason why exposed patients experience CTS-like symptoms or have a propensity to develop CTS [3]. An increased occurrence of osteoarthritis in joints of the hand related to vibration exposure has been suggested [13]. A less highlighted condition, although questioned to be work-related [14], and linked to exposure to vibrating hand-held tools is Dupuytren's disease with finger contracture affecting the palmar fascia and bending of fingers [8]. However, a genetic component [15] as well as a relation to diabetes and occupation [16] may be more common causes of Dupuytren´s disease. Vibration exposure may promote or speed up the development of Dupuytren's contracture in a genetically predisposed individual.

For peer review only - http://bmjopen.bmj.com/site/about/guidelines.xhtml

Most previous studies report on one of the hand and forearm conditions, and few have reported the occurrence of all conditions suggested to be related to vibration exposure. We aimed to analyze the presence of five common hand and forearm disorders in a large population-based cohort, where a proportion of the individuals reported that they were “not at all” exposed or exposed to vibrating hand-held tools to “some” or “much” extent.

METHODS

Study population

The population-based register from southern Sweden, the Malmö Diet and Cancer Study (MDCS), which has previously been described [17, 18], was used in this observational study. In brief, 30,446 individuals aged 46-73 were invited to participate between 1991-1996. Initially, individuals were screened for cardiovascular risk factors, undergoing clinical examination and laboratory assessment under fasting conditions. In addition, they responded to several questionnaires. One of the questionnaires contained the question, *“Does your work involve working with vibrating hand-held tools?”* where the individuals responded, *“Not at all”*, *“Some”* and *“Much”*. This questionnaire was answered by 14,342 individuals included in the present study. Hence, we report self-reported vibration exposure. In addition, other relevant variables were noted, including smoking, alcohol consumption, hypertension, and diabetes mellitus. Smoking was self-reported at baseline. Alcohol consumption was self-reported use during the last week at baseline and reported as g/day. Diabetes data was obtained through linkage with the National Diabetes Register (NDR), which contains data from both hospital care and primary health care on patients with diabetes. The present or latest job was defined according to the Swedish socioeconomic classification into three groups; employees (including officials/salaried employees), laborers (including laborers in the production of goods or services as well as farmers), or self-employed. Non-participants of the

For peer review only

MDCS are best described in the study by Manjer et al [18], where mortality was higher in non-participants, which might reflect a larger disease burden and less willingness to participate.

Endpoint retrieval

In the present study, we used data from national registers managed by the National Board of Health and Welfare to identify whether the included individuals had been treated for the hand and forearm conditions of interest. The used registers include data from hospitals and specialized care settings (outpatient clinics) but not from primary care units. Inpatient care has been registered since 1964, outpatient surgeries are included from 1997, and visits in specialized outpatient clinics are included from 2001. The included hand and forearm conditions were the two most common nerve compression disorders [carpal tunnel syndrome (ICD-10 code G56.0) and ulnar nerve entrapment at the elbow or the wrist (G56.2)], Dupuytren's disease with contracture (M72.0), trigger finger (M65.3) and osteoarthritis of the first CMC joint (M18.X). Patients were followed until death, emigration, or end of study 2018-12-31, whichever occurred first.

Statistical analysis

The baseline characteristics are presented as median (25th to 75th percentiles) or proportion of patients n (%). Since the continuous variables (age and alcohol consumption) were not normally distributed, we used non-parametric testing. The Kruskal-Wallis test was used to detect any statistical significance between groups based on vibration exposure for continuous variables. If p-value <0.05, a Mann-Whitney U-test was used for pairwise individual comparisons between the three groups. For ordinal variables, the Chi^2-test was used for group comparisons. Cox regression analysis was performed to obtain Hazard Ratios (HR) for risk of

For peer review only - http://bmjopen.bmj.com/site/about/guidelines.xhtml

development of hand and forearm pathology due to vibration exposure. Time to event was defined as follow-up period from screening to first disease event or to last follow-up date (2018-12-31; years), emigration or death. In the group with "much" vibration exposure, there were 82% men, hence the results were stratified by sex. The group without vibration exposure was used as a reference group. We also analyzed vibration exposure as a dichotomous variable ("no" vibration exposure vs. "any" vibration exposure). The first models were unadjusted. The second models were adjusted for age at baseline, sex, prevalent diabetes before the study started, smoking, hypertension, and alcohol consumption. Confounders were chosen based on previous data and the authors' clinical experience on influencing factors for the specific diagnosis [19-21] (see also Discussion). Finally, Kaplan Meier curves were used to visualize disease-free survival. All statistical analysis was made using IBM SPSS Statistics for Mac version 28 (SPSS Inc., Chicago, IL, USA), and a two-sided p-value <0.05 was considered significant.

Patient and Public Involvement Statement

No patients, politicians, or the public were involved in elaborating on the present research question or the design of the study. The obtained results, after publication in the scientific journal, will be disseminated in different ways.

RESULTS

Characteristics

In total, 12,220/14,342 individuals (85%) reported that they did not have had "any" exposure to vibrating hand-held tools, while 1,392/14,342 (10%) reported that they had "some" and 730/14,342 (5%) had "much" exposure to vibrating hand-held tools. Age was median 57±6 years in all groups. There were more men in the vibration exposed groups ["not at all"

For peer review only - http://bmjopen.bmj.com/site/about/guidelines.xhtml

4,845/12,220 (40%) vs 1,003/1,392 (72%) in the "some" group and 601/730 (82%) in the "much" group]. Most included individuals (7181/14,342; 50%) were employees, 5,440/14,342 (38%) were laborers, and 1,684/14,342 (12%) were self-employed. Occupational data was missing in 37 individuals. Basic characteristics are presented in **Supplemental table 1**. Some differences in basic characteristics were observed among men and women related to the extent of reported vibration exposure (**Table 1**).

Carpal tunnel syndrome and ulnar nerve compression at the elbow or wrist

Among the men, CTS and UNE were more common in the groups with more vibration exposure; however, not being statistically significant for CTS (**Table 1**). In the Cox regression analysis, "much" vibration exposure was independently associated with CTS [Hazard Ratio 1.71 (95% CI 1.11-2.62] and UNE [HR 2.42 (1.15-5.07); **Table 2**]. "Any" vibration exposure was independently associated with a greater risk of CTS [HR 1.41 (1.02-1.95); **Table 2**]. "Some" vibration exposure was independently associated with UNE [HR 2.10 (1.12-3.95); **Table 2**]. Among the women, CTS seemed more common in the group without vibration exposure, however, it was not statistically significant. The Cox regression models for women showed no significant effects of vibration exposure on the risk of developing CTS and UNE (for survival analysis using Kaplan Meier curves in men and women regarding CTS and UNE see **Figures 1A -1D)**.

For peer review only

For peer review only - http://bmjopen.bmj.com/site/about/guidelines.xhtml

Table 1. Baseline characteristics of individuals that reported exposure to vibrating hand-held tools as "not at all", "some" and "much" stratified by sex.

	"Does your work involve working with vibrating hand-held tools?"							
	Men (n=6448)				Women (n=7892)			
	**"Not at all" n=4,845**	**"Some" n=1,003**	**"Much" n=601**	**P-value**	**"Not at all" n=7,374**	**"Some" n=389**	**"Much" n=129**	**P-value**
Age (years)	57±6	57±6	57±6	0.67	57±6	58±6	57±6	0.079
Current smoking	1,355 (28%)	355 (35%)	211 (35%)	**<0.001**†,‡	2,103 (29%)	122 (31%)	46 (36%)	0.10
Antihypertensive treatment	2,499 (52%)	529 (53%)	329 (55%)	0.306	3,264 (44%)	179 (46%)	44 (34%)	0.053
Prevalent diabetes	269 (6%)	69 (7%)	33 (6%)	0.249	277 (4%)	16 (4%)	4 (3%)	0.87
Alcohol consumption (g/day)	12.2 [4.5-23.0]	9.6 [2.9-21.3]	10.6 [1.8-20.1]	**<0.001**†,‡	5.4 [0.8-11.2]	4.4 [0.46-10.0]	4.1 [0.0-10.3]	**0.019**†

For peer review only - http://bmjopen.bmj.com/site/about/guidelines.xhtml

Laborers (n, %)	1234 (25%)	630 (63%)	472 (79%)	**<0.001**†,‡	2764 (37%)	263 (68%)	77 (60%)	**<0.001**†,‡
Incident carpal tunnel syndrome (CTS)	133 (2.7%)	34 (3.4%)	26 (4.3%)	0.072	327 (4.4%)	9 (2.3%)	3 (2.3%)	0.071
Incident ulnar nerve entrapment at elbow/wrist (UNE)	33 (0.7%)	14 (1.4%)	9 (1.5%)	**0.019**‡	51 (0.7%)	2 (0.5%)	2 (1.6%)	0.46
Incident Dupuytren's contracture	158 (3.3)	25 (2.5%)	16 (2.7%)	0.36	87 (1.2)	7 (1.8%)	2 (1.6%)	0.52
Incident trigger finger	137 (2.8%)	28 (2.8%)	23 (3.8%)	0.38	262 (3.6%)	13 (3.3%)	11 (8.5%)	**0.011**‡,§
Incident osteoarthritis of the thumb CMC-1 joint	61 (1.3%)	19 (1.9%)	11 (1.8%)	0.20	236 (3.2%)	14 (3.6%)	6 (4.7%)	0.60
Any incident hand and forearm disorder	442 (9.1%)	94 (9.4%)	69 (11.5%)	0.17	793 (10.8%)	41 (10.5%)	18 (14%)	0.50

For peer review only - http://bmjopen.bmj.com/site/about/guidelines.xhtml

P-value for good comparisons between the subjects are based on Kruskal Wallis and Mann-Whitney U-test as a posthoc test and Chi2 test for ordinal variables. † Statistical significance was found between "not at all" and "some". ‡ Statistical significance was found between "not at all" and "much". § Statistical significance found between "some" and "much".

For peer review only

Table 2. Cox regression models – unadjusted and adjusted for relevant factors – with the association between vibration exposure and risk for development of five specific hand and forearm conditions and any of the hand and forearm conditions stratified by sex.

**Men**						
Model 1 - unadjusted						
	**CTS**	**UNE**	**Trigger finger**	**Dupuytren's contracture**	**CMC-1 osteoarthritis**	**Any hand and forearm condition**
"No" vibration exposure	*Reference*					
"Any" vibration exposure	1.43 (1.04-1.97)	1.18 (0.69-2.04)	1.22 (0.88-1.70)	1.10 (0.78-1.57)	1.27 (0.82-1.97)	1.30 (1.08-1.55)
"Some" vibration exposure	1.21 (0.82-1.79)	2.20 (1.17-4.12)	1.01 (0.67-1.53)	0.78 (0.51-1.20)	1.57 (0.94-2.64)	1.05 (0.84-1.32)
"Much" vibration exposure	1.71 (1.11-2.62)	2.44 (1.17-5.12)	1.50 (0.97-2.34)	0.90 (0.54-1.50)	1.55 (0.82-2.95)	1.40 (1.08-1.80)
Model 2 – adjusted for age, smoking, alcohol consumption, hypertension and diabetes						
"No" vibration exposure	Reference					
"Any" vibration exposure	1.41 (1.02-1.95)	0.84 (0.44-1.60)	1.22 (0.88-1.70)	1.15 (0.81-1.65)	1.56 (0.97-2.50)	1.33 (1.10-1.60)
"Some" vibration exposure	1.18 (0.80-1.76)	2.10 (1.12-3.95)	1.01 (0.67-1.53)	0.82 (0.53-1.27)	1.55 (0.92-2.61)	1.06 (0.84-1.33)
"Much" vibration exposure	1.71 (1.11-2.62)	2.42 (1.15-5.07)	1.54 (0.99-2.39)	0.96 (0.57-1.61)	1.55 (0.81-2.94)	1.44 (1.12-1.86)

For peer review only - http://bmjopen.bmj.com/site/about/guidelines.xhtml

**Women**						
Model 1 - unadjusted						
	**CTS**	**UNE**	**Trigger finger**	**Dupuytren's contracture**	**CMC-1 osteoarthritis**	**Any hand and forearm condition**
"No" vibration exposure	*Reference*					
"Any" vibration exposure	0.85 (0.48-1.52)	0.89 (0.32-2.50)	1.08 (0.71-1.64)	0.85 (0.43-1.70)	2.06 (1.29-3.27)	1.12 (0.86-1.46)
"Some" vibration exposure	0.56 (0.29-1.08)	0.83 (0.20-3.40)	1.02 (0.59-1.78)	1.67 (0.77-3.60)	1.20 (0.70-2.06)	1.06 (0.77-1.45)
"Much" vibration exposure	0.56 (0.18-1.75)	2.50 (0.61-10.26)	2.72 (1.49-4.96)	1.43 (0.35-5.83)	1.55 (0.69-3.48)	1.43 (0.89-2.28)
Model 2 – adjusted for age, smoking, alcohol consumption, hypertension, and diabetes						
"No" vibration exposure	Reference					
"Any" vibration exposure	0.86 (0.48-1.54)	0.78 (0.25-2.44)	1.06 (0.70-1.63)	0.71 (0.34-1.51)	1.96 (1.23-3.12)	1.09 (0.83-1.42)
"Some" vibration exposure	0.56 (0.29-1.09)	0.82 (0.20-3.36)	1.05 (0.60-1.83)	1.76 (0.81-3.80)	1.19 (0.69-2.03)	1.07 (0.78-1.46)
"Much" vibration exposure	0.56 (0.18-1.76)	2.54 (0.62-10.48)	2.73 (1.49-4.99)	1.57 (0.39-6.38)	1.57 (0.70-3.54)	1.45 (0.91-2.32)

CTS; carpal tunnel syndrome, UNE; ulnar nerve entrapment at elbow/wrist, CMC-1; first carpometacarpal joint

For peer review only - http://bmjopen.bmj.com/site/about/guidelines.xhtml

Figure 1. Survival analysis using Kaplan Meier curves of the studied hand and forearm conditions that were followed from screening to the first disease event or to the last follow-up date (Dec 31^{st,} 2018) and were potentially associated with vibration exposure stratified by sex. The individuals replied to the question "Does your work involve working with vibrating hand-held tools?" with possible answers "Not at all", "Some" and "Much".

Trigger finger

Trigger finger was more common among women with "much" vibration exposure than women with "some" or "no" vibration exposure (**Table 1**). No differences could be seen between the groups of men. "Much" vibration exposure was independently associated with the development of trigger finger in women [HR 2.72 (1.49-4.96)], but not in men (**Table 2;** for survival analysis using Kaplan Meier curves **see Figures 1E -1F**).

Dupuytren's contracture

Dupuytren's contracture was equally common among the groups in both sexes, and no significant effect of vibration exposure on the development of Dupuytren's disease with contracture could be demonstrated in the Cox regression analysis (**Table 1** and **2;** for survival analysis using Kaplan Meier curves **see Figures 1G - 1H**).

Osteoarthritis of the first CMC joint

CMC-1 OA was equally common among the groups in both sexes. In women, "any" vibration exposure was independently associated with the risk of CMC-1 OA [HR 1.96 (1.23-3.12)] in the Cox regression analysis (**Table 1** and **2;** for survival analysis using Kaplan Meier curves **see Figures 1I - 1J**).

For peer review only

Any hand and forearm condition

We found similar proportions of any incident hand and forearm condition among all groups (**Table 1**). In the Cox regression analysis, “much” vibration exposure predicted any hand and forearm condition in men [HR 1.44 (1.12-1.86)] but not in women [HR 1.45 (0.91-2.32)] (**Table 2;** for survival analysis using Kaplan Meier curves **see Figures 1K -1L**). “Any” vibration exposure was associated with a greater risk of any hand and forearm condition in men [HR 1.33 (1.10-1.60) (**Table 2**).

DISCUSSION

The present observational study of a large population-based cohort, where the individuals replied to a question concerning exposure to vibrating hand-held tools and graded as “not at all”, “some” and “much”, shows that hand and forearm conditions in men, such as CTS and UNE, were significantly higher than in un-exposed men, particularly among those with “much” vibration exposure when adjusted for several relevant confounding factors. In addition, any hand and forearm condition were associated with “any” and “much” exposure to vibrating hand-held tools. In contrast, in women, there was an association between “any” exposure to vibrating hand-held tools and CMC-1 OA, and an association between “much” vibration exposure and trigger finger. The presently described hand and forearm conditions are common in the general population, irrespective of vibration exposure, where prevalence figures for CTS, UNE, Dupuytren´s contracture, trigger finger, and osteoarthritis of the CMC-1 joint are reported as 1.4-15 % [20, 22, 23]. Previously published data has mainly included one of the present diagnoses at a time associated to vibration exposure, while the data from the current cohort also allowed the inclusion of all the relevant, and previously discussed,

For peer review only - http://bmjopen.bmj.com/site/about/guidelines.xhtml

conditions in the hand and forearm associated to exposure to vibrating hand-held tools [8, 24-26].

Risk assessment in vibration exposure of hand-held tools to different hand and forearm disorders may be complex due to the high prevalence of some of the hand and forearm disorders as well as the association with relevant factors and general comorbidities that must be adjusted for in any statistical analysis [8, 13, 21, 26, 27]. In this study, we stratified our data on sex due to the relatively high number of male individuals in the cohort, as well attempting to level out any difference in occurrence of vibration exposure in men and women as sex is known to influence the prevalence of hand and forearm diagnoses [28]. In our *Model 2*, we adjusted for factors that may be of relevance in development of the included hand and forearm disorders, i.e., age, smoking, alcohol consumption, hypertension, and diabetes; variables that are accessible in the MDCS database [19, 27]. Age is a highly relevant influencing factor for development hand and forearm disorders, where men and women have different age distributions for development of CTS and UNE as well as of osteoarthritis in the first CMC joint [23, 28, 29]. In contrast to our study, other authors have reported an increased risk for CTS in both sexes, although being highest among young men [24], which may be explained by differences between the cohorts and adjustment of confounding factors. Smoking may affect both the development and outcome of surgery of CTS and UNE [27], due to its effect on the intraneural microcirculation as well as being highlighted in Dupuytren´s disease with contracture [30]. Individuals with hypertension may also have a higher perfusion pressure in the intraneural blood vessels making the nerve more resistant to compression [31]; again, indicating that adjustment for several factors in the Cox analysis is necessary [32]. Development of CTS, UNE, Dupuytren´s disease with finger contracture, and trigger fingers are also associated with both type 1 and type 2 diabetes [19], which is probably not entirely

For peer review only - http://bmjopen.bmj.com/site/about/guidelines.xhtml

true for osteoarthritis of the first CMC joint, for which high BMI and obesity are significant risk markers/factors [33]. Finally, alcohol consumption is also a factor that may be involved in development of Dupuytren´s contracture as well as a cause of UNE [19, 34]. Thus, it is crucial to adjust for age, smoking, alcohol consumption, hypertension, and diabetes when analyzing the risk of developing various hand and forearm disorders in relation to vibration exposure of hand-held tools. We also included the association of any hand and forearm disorder as a variable in relation to vibration exposure, which is appropriate since there usually is an extensive co-morbidity of various hand and forearm disorders, such as concomitant CTS in surgically treated patients with UNE, as well as the other present hand and forearm conditions [35]. In addition, a subject with diabetes, irrespective of having type 1 or type 2 diabetes or if neuropathy is present, may have an increased susceptibility to a nerve compression disorders as a subject with a history of vibration exposure.

The observed differences in risks for developing the studied hand and forearm conditions in men and women are interesting and may have different explanations. Surprisingly, in women, “much” vibration exposure only increased the risk of trigger finger, while in men, the risks were higher for CTS and UNE. Women also differed in the marked risk for CMC-1 OA with “any” vibration exposure. There are no apparent reasons for this discrepancy. Still, it has been suggested that an underlying neuropathy may be important for development of CTS in vibration-exposed individuals [3, 8, 12]. One other study, however, found that working with vibrating tools for more than two hours per day was a risk factor for developing CTS in women but not in men [36]. Furthermore, neuropathy is reported to be more common and more severe in men with diabetes [24, 37]. Therefore, one may speculate that similar mechanisms may explain the present sex difference concerning nerve compression disorders. Trigger finger is more common among women, and it has been speculated that estrogen

For peer review only - http://bmjopen.bmj.com/site/about/guidelines.xhtml

deficiency alters tendon metabolism in postmenopausal women, hence impacting the risk of developing trigger finger [20]. Previous studies have failed to demonstrate an association between occupational factors and trigger finger [20]. It is, however, possible that during the early 1990s, when this cohort study started, occupational sex differences of risk existed, and among the participants who worked with vibrating hand-held tools, men worked with large tools, such as grinding machines and chainsaws. In contrast, women may have worked more with precision dental drills and other smaller tools. Whether the type of tool affects these disorders' development is unknown.

Our data also indicates that there is a greater risk of osteoarthritis in the first CMC joint in vibration-exposed women. In contrast, one older study investigated radiological osteoarthritis in vibration exposed and non-exposed individuals, and found no significant differences between groups, indicating that the development of such a condition has other causes than vibration exposure alone [25]. It is, however, unclear whether that study included any women. One more recent meta-analysis could not demonstrate any increased risk of hand OA from vibration exposure [13]. However, women have been underrepresented in many studies on vibration exposure. There may also be a possibility that women may be exposed to other magnitudes and frequencies of vibrations like from various precision instruments requiring a firm pinch grip, such as dental instruments, as indicated by the present higher proportion of laborers among men with “much” vibration exposure compared to the lower proportion of laborers among women with “much” vibration exposure [38-41]. Tasks requiring pinch strength of higher magnitude have been associated with an increased risk of hand OA [13].

It was not possible and was not the present study’s purpose to evaluate any surgery outcome for the conditions in the individuals with “no”, “some”, or “much” exposure to vibration. The

For peer review only - http://bmjopen.bmj.com/site/about/guidelines.xhtml

result of surgery in vibration-exposed patients with CTS seems to be impaired compared to otherwise healthy patients with CTS [42]. Still, outcome data are essentially lacking for the other hand and forearm conditions included in the present study, except for Dupuytren´s contracture, for which socioeconomic status has to be considered [43]. Earlier exposure to vibrating tools may affect the pre- and postoperative function in individuals undergoing surgery for Dupuytren's contracture. However, the satisfaction or return to work was not different [44]. Hence, whether vibration exposure affects clinical outcomes following treatment for common hand and forearm conditions remains an important area for future research.

Limitations

There are some limitations of the study, such as that in >10 % of the individuals, no information was available concerning vibration exposure as well as a 41% response rate among the population-based cohort [18]. This might potentially limit the generalizability of this study and there might exist a selection bias in who answered the questionnaires. However, based on previous information there may be an age difference (i.e., younger being non-responders) between those who do respond and do not respond to questionnaires [45, 46], but recent data indicate that the response rate may not be relevant [47]. In addition, our diagnoses were based on ICD-codes from the National Board of Health and Welfare, where both surgically and not surgically treated cases were included, although without any patients diagnosed in primary care. This might include a selection bias, with a possible underestimation of the prevalence since patients with milder forms of the studied hand and forearm conditions that can be treated conservatively are managed in primary care. Quantifying the vibration exposure is relevant but is not possible in larger cohorts as in the present study, which is a limitation. We also had no data on hand dominance, nor ethnicity

For peer review only

and socioeconomic deprivation that might influence prevalence of the studied conditions. Access to socioeconomic data may have sub-analyses related to different socioeconomic status to be performed. Thus. the present prevalence may be underestimated if only those with manual work of certain severity, low income and low education levels are evaluated. However, analysis of socioeconomic status in relation to vibration exposure was not in the scope of the study but is a future project.

CONCLUSIONS

We conclude that vibration exposure to hand-held tools increases the risk of having or developing CTS, and particularly UNE, but also any common hand and forearm conditions in men and CMC-1 OA and trigger finger in women. It is essential to adjust for relevant influencing factors, such as age, smoking, alcohol consumption, hypertension, and diabetes, when investigating the risks of working with hand-held vibrating tools.

STATEMENTS

Author contributions

M.Z., P.N., and L.B.D. conceived the ideas; M.Z. and P.N. collected the data; M.Z., M.R., and L.B.D. analyzed the data; M.Z. and L.B.D. led the writing, and all authors contributed to the interpretation and the writing and approved the final version of the manuscript.

Funding statement

This research was funded by the Swedish Research Council (#2022-01942, principal investigator LBD), the Swedish Diabetes Foundation (#DIA2020-492, principal investigator LBD), the Regional Agreement on Medical Training and Clinical Research (ALF) between Region Skåne and Lund University (#2018-Projekt 0104; PI LBD) and Funds from Skåne

For peer review only - http://bmjopen.bmj.com/site/about/guidelines.xhtml

University Hospital (#2019-659; PI LBD), Elly Olsson´s Foundation for scientific research (#Not applicable), Stig and Ragna Gorthon Foundation (#Not applicable), Almroth Foundation (#Not applicable), Kockska foundation (#Not applicable), and the Magnus Bergvall Foundation [#2020-03612, principal investigator MZ].

The initial financial support to the Malmö Diet and Cancer Study cohort at baseline was provided by the Swedish Cancer Foundation (#Not applicable), The Swedish Medical Research Council (#Not applicable), The European Commission (#Not applicable), the City of Malmö (#Not applicable), the Swedish Dairy Association (#Not applicable) and the Albert Påhlsson Foundation (#Not applicable).

The sponsors had no role in the study design, collection, analysis, or interpretation of the data, writing the report, or decision to submit the manuscript.

Competing of Interests

The authors declare no conflict of interest.

Ethics Approval

Participants provided written informed consent before inclusion in MDCS. MDCS and the present study have been approved by the regional Ethics committee in Lund and the Swedish Ethical Review Authority (DNR: LU51-90; 2009-633; 2019-01439). The study was conducted in accordance with the Helsinki Declaration.

Data Sharing

The data are not publicly available due to ethical restrictions. The data that support the findings of this study are only available on request from the corresponding author as well as after application and approval by the Swedish Ethical Review Authority (https://etikprovningsmyndigheten.se/en/ due to the restrictions in Sweden, based on the

For peer review only - http://bmjopen.bmj.com/site/about/guidelines.xhtml

national Law for ethical review of research on humans [“Lag (2003:460) om etikprövning av forskning som avser människor”].

Acknowledgments

The authors are very grateful to all the participants in MDCS whose participation enabled this work. We are also thankful to the National Diabetes Register (NDR) and Statistics Sweden (SCB) for register data, Anders Dahlin for his help with data extraction, and Tina Folker for her administrative assistance. We acknowledge support from the Lund University Infrastructure grant ”Malmö population-based cohorts” (STYR 2019/2046).

References

1. Dong RG, Rakheja S, Schopper AW, et al. Hand-transmitted vibration and biodynamic response of the human hand-arm: a critical review. *Crit Rev Biomed Eng* 2001;29(4):393-439. doi: 10.1615/critrevbiomedeng.v29.i4.20
2. Pelmear P, Leong D. Review of occupational standards and guidelines for hand-arm (segmental) vibration syndrome (HAVS). *Appl Occup Environ Hyg* 2000;15(3):291-302.
3. Dahlin LB, Sanden H, Dahlin E, et al. Low myelinated nerve-fibre density may lead to symptoms associated with nerve entrapment in vibration-induced neuropathy. *J Occup Med Toxicol* 2014;9(1):7. doi: 10.1186/1745-6673-9-7
4. Lundborg G, Dahlin LB, Hansson H-A, et al. Vibration exposure and peripheral nerve fiber damage. *J Hand Surg* 1990;15A:346-51.
5. Hulkkonen S, Shiri R, Auvinen J, et al. Risk factors of hospitalization for carpal tunnel syndrome among the general working population. *Scand J Work Environ Health* 2020;46(1):43-49. doi: 10.5271/sjweh.3835 [published Online First: 20190606]

For peer review only

6. Lawson IJ. Is carpal tunnel syndrome caused by work with vibrating tools? *Occup Med (Lond)* 2020;70(1):8-10. doi: 10.1093/occmed/kqz142

7. Piligian G, Herbert R, Hearns M, et al. Evaluation and management of chronic work-related musculoskeletal disorders of the distal upper extremity. *Am J Ind Med* 2000;37(1):75-93. doi: 10.1002/(sici)1097-0274(200001)37:1<75::aid-ajim7>3.0.co;2-4

8. Mathieu S, Naughton G, Descatha A, et al. Dupuytren's Disease and exposure to vibration: Systematic review and Meta-analysis. *Joint Bone Spine* 2020;87(3):203-07. doi: 10.1016/j.jbspin.2020.02.001 [published Online First: 20200212]

9. Aarhus L, Stranden E, Nordby KC, et al. Vascular component of hand-arm vibration syndrome: a 22-year follow-up study. *Occup Med (Lond)* 2018;68(6):384-90. doi: 10.1093/occmed/kqy085

10. Wei N, Yan R, Lang L, et al. Local vibration induced vascular pathological structural changes and abnormal levels of vascular damage indicators. *Microvasc Res* 2021;136:104163. doi: 10.1016/j.mvr.2021.104163 [published Online First: 20210406]

11. Necking LE, Lundborg G, Lundstrom R, et al. Hand muscle pathology after long-term vibration exposure. *The Journal of Hand Surgery British Volume* 2004;29(5):431-7. doi: 10.1016/j.jhsb.2004.05.004

12. Stromberg T, Dahlin LB, Brun A, et al. Structural nerve changes at wrist level in workers exposed to vibration. *Occup Environ Med* 1997;54(5):307-11.

13. Hammer PE, Shiri R, Kryger AI, et al. Associations of work activities requiring pinch or hand grip or exposure to hand-arm vibration with finger and wrist osteoarthritis: a meta-analysis. *Scand J Work Environ Health* 2014;40(2):133-45. doi: 10.5271/sjweh.3409 [published Online First: 2013/12/07]

For peer review only

For peer review only - http://bmjopen.bmj.com/site/about/guidelines.xhtml

14. Descatha A, Jauffret P, Chastang JF, et al. Should we consider Dupuytren's contracture as work-related? A review and meta-analysis of an old debate. *BMC Musculoskelet Disord* 2011;12:96. doi: 10.1186/1471-2474-12-96 [published Online First: 2011/05/18]

15. Ng M, Thakkar D, Southam L, et al. A Genome-wide Association Study of Dupuytren Disease Reveals 17 Additional Variants Implicated in Fibrosis. *Am J Hum Genet* 2017;101(3):417-27. doi: 10.1016/j.ajhg.2017.08.006

16. van den Berge BA, Wiberg A, Werker PMN, et al. Dupuytren's disease is a work-related disorder: results of a population-based cohort study. *Occup Environ Med* 2023;80(3):137-45. doi: 10.1136/oemed-2022-108670 [published Online First: 20230112]

17. Berglund G, Elmstahl S, Janzon L, et al. The Malmo Diet and Cancer Study. Design and feasibility. *J Intern Med* 1993;233(1):45-51. doi: 10.1111/j.1365-2796.1993.tb00647.x [published Online First: 1993/01/01]

18. Manjer J, Carlsson S, Elmstahl S, et al. The Malmo Diet and Cancer Study: representativity, cancer incidence and mortality in participants and non-participants. *Eur J Cancer Prev* 2001;10(6):489-99. doi: 10.1097/00008469-200112000-00003 [published Online First: 2002/03/28]

19. Rydberg M, Zimmerman M, Lofgren JP, et al. Metabolic factors and the risk of Dupuytren's disease: data from 30,000 individuals followed for over 20 years. *Sci Rep* 2021;11(1):14669. doi: 10.1038/s41598-021-94025-7 [published Online First: 2021/07/21]

20. Okita G, Hayashi M, Ikegami S, et al. The Prevalence and Risk Factors for Trigger Digits in a Random Sampling of a Japanese Population Registry between 50 and 89 Years of

For peer review only

For peer review only - http://bmjopen.bmj.com/site/about/guidelines.xhtml

Age. *J Hand Surg Asian Pac Vol* 2022;27(1):148-55. doi: 10.1142/S2424835522500151 [published Online First: 20220209]

21. Jones G, Cooley HM, Stankovich JM. A cross sectional study of the association between sex, smoking, and other lifestyle factors and osteoarthritis of the hand. *J Rheumatol* 2002;29(8):1719-24.
22. Haara MM, Heliovaara M, Kroger H, et al. Osteoarthritis in the carpometacarpal joint of the thumb. Prevalence and associations with disability and mortality. *J Bone Joint Surg Am* 2004;86(7):1452-7. doi: 10.2106/00004623-200407000-00013
23. Atroshi I, Gummesson C, Johnsson R, et al. Prevalence of carpal tunnel syndrome in a general population [see comments]. *JAMA* 1999;282(2):153-58.
24. Vihlborg P, Pettersson H, Makdoumi K, et al. Carpal Tunnel Syndrome and Hand-Arm Vibration: A Swedish National Registry Case-Control Study. *J Occup Environ Med* 2022;64(3):197-201. doi: 10.1097/JOM.0000000000002451
25. Malchaire J, Maldague B, Huberlant JM, et al. Bone and joint changes in the wrists and elbows and their association with hand and arm vibration exposure. *Ann Occup Hyg* 1986;30(4):461-8. doi: 10.1093/annhyg/30.4.461
26. van Rijn RM, Huisstede BM, Koes BW, et al. Associations between work-related factors and the carpal tunnel syndrome--a systematic review. *Scand J Work Environ Health* 2009;35(1):19-36. doi: 10.5271/sjweh.1306
27. Pourmemari MH, Viikari-Juntura E, Shiri R. Smoking and carpal tunnel syndrome: a meta-analysis. *Muscle Nerve* 2014;49(3):345-50. doi: 10.1002/mus.23922 [published Online First: 20130708]
28. Rydberg M, Zimmerman M, Gottsater A, et al. Diabetic hand: prevalence and incidence of diabetic hand problems using data from 1.1 million inhabitants in southern Sweden.

For peer review only

BMJ Open Diabetes Res Care 2022;10(1) doi: 10.1136/bmjdrc-2021-002614 [published Online First: 2022/01/21]

29. Mitake T, Iwatsuki K, Hirata H. Differences in characteristics of carpal tunnel syndrome between male and female patients. *J Orthop Sci* 2020;25(5):843-46. doi: 10.1016/j.jos.2019.10.017 [published Online First: 20191125]

30. Eckerdal D, Nivestam A, Dahlin LB. Surgical treatment of Dupuytren's disease - outcome and health economy in relation to smoking and diabetes. *BMC Musculoskelet Disord* 2014;15:117. doi: 10.1186/1471-2474-15-117 [published Online First: 20140402]

31. Szabo RM, Gelberman RH, Williamson RV, et al. Effects of increased systemic blood pressure on the tissue fluid pressure threshold of peripheral nerve. *J Orthop Res* 1983;1(2):172-8. doi: 10.1002/jor.1100010208

32. Mustafa KN, Khader YS, Bsoul AK, et al. Musculoskeletal disorders of the hand in type 2 diabetes mellitus: prevalence and its associated factors. *Int J Rheum Dis* 2016;19(7):730-5. doi: 10.1111/1756-185X.12617

33. Rydberg M, Dahlin LB, Gottsater A, et al. High body mass index is associated with increased risk for osteoarthritis of the first carpometacarpal joint during more than 30 years of follow-up. *RMD Open* 2020;6(3) doi: 10.1136/rmdopen-2020-001368 [published Online First: 2020/10/29]

34. Nordenskjold J, Englund M, Zhou C, et al. Prevalence and incidence of doctor-diagnosed Dupuytren's disease: a population-based study. *J Hand Surg Eur Vol* 2017;42(7):673-77. doi: 10.1177/1753193416687914

35. Giostad A, Nyman E. Patient Characteristics in Ulnar Nerve Compression at the Elbow at a Tertiary Referral Hospital and Predictive Factors for Outcomes of Simple Decompression versus Subcutaneous Transposition of the Ulnar Nerve. *Biomed Res*

For peer review only

For peer review only - http://bmjopen.bmj.com/site/about/guidelines.xhtml

Int 2019;2019:5302462. doi: 10.1155/2019/5302462 [published Online First: 2020/01/18]

36. Melchior M, Roquelaure Y, Evanoff B, et al. Why are manual workers at high risk of upper limb disorders? The role of physical work factors in a random sample of workers in France (the Pays de la Loire study). *Occup Environ Med* 2006;63(11):754-61. doi: 10.1136/oem.2005.025122 [published Online First: 20060620]

37. Kamenov ZA, Parapunova RA, Georgieva RT. Earlier development of diabetic neuropathy in men than in women with type 2 diabetes mellitus. *Gend Med* 2010;7(6):600-15. doi: 10.1016/j.genm.2010.11.001

38. Bylund SH, Burstrom L, Knutsson A. A descriptive study of women injured by hand-arm vibration. *Ann Occup Hyg* 2002;46(3):299-307. doi: 10.1093/annhyg/mef025

39. Nordander C, Ohlsson K, Balogh I, et al. Gender differences in workers with identical repetitive industrial tasks: exposure and musculoskeletal disorders. *Int Arch Occup Environ Health* 2008;81(8):939-47. doi: 10.1007/s00420-007-0286-9 [published Online First: 20071208]

40. Neely G, Burstrom L. Gender differences in subjective responses to hand–arm vibration. *International Journal of Industrial Ergonomics* 2006;36(2):135-40.

41. Biswas A, Harbin S, Irvin E, et al. Sex and Gender Differences in Occupational Hazard Exposures: a Scoping Review of the Recent Literature. *Curr Environ Health Rep* 2021;8(4):267-80. doi: 10.1007/s40572-021-00330-8 [published Online First: 20211127]

42. Stirling PHC, Jenkins PJ, Clement ND, et al. The Influence of Self-Reported Hand-Arm Vibration Exposure on Functional Outcomes Following Carpal Tunnel Release. *J Hand Surg Am* 2020;45(11):1029-36. doi: 10.1016/j.jhsa.2020.08.018

For peer review only

43. Stirling PHC, Jenkins PJ, McEachan JE. Previous vibration exposure in patients undergoing surgical treatment of Dupuytren's contracture. *J Hand Surg Eur Vol* 2020;45(5):525-27. doi: 10.1177/1753193420903675 [published Online First: 20200130]

44. Stirling PHC, Ng N, Jenkins PJ, et al. Hand-arm vibration and outcomes of surgery for Dupuytren's contracture. *Occup Med (Lond)* 2021;71(4-5):219-22. doi: 10.1093/occmed/kqab070

45. Zimmerman M, Eeg-Olofsson K M.D P, Svensson Am Rn P, et al. Open carpal tunnel release and diabetes: a retrospective study using PROMs and national quality registries. *BMJ Open* 2019;9(9):e030179. doi: 10.1136/bmjopen-2019-030179 [published Online First: 2019/09/07]

46. Frostadottir D, Ekman L, Zimmerman M, et al. Cold sensitivity and its association to functional disability following a major nerve trunk injury in the upper extremity-A national registry-based study. *PLoS One* 2022;17(7):e0270059. doi: 10.1371/journal.pone.0270059 [published Online First: 2022/07/13]

47. Stirling PHC, Jenkins PJ, Ng N, et al. Nonresponder bias in hand surgery: analysis of 1945 cases lost to follow-up over a 6-year period. *J Hand Surg Eur Vol* 2022;47(2):197-205. doi: 10.1177/17531934211045627 [published Online First: 2021/09/17]

For peer review only - http://bmjopen.bmj.com/site/about/guidelines.xhtml

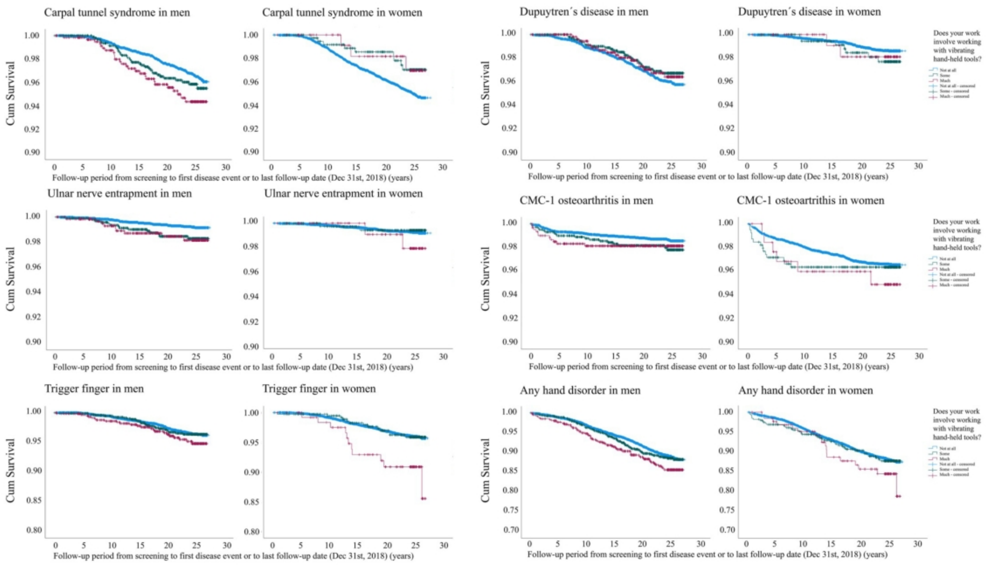

440x254mm (300 x 300 DPI)

Supplemental table 1. Baseline characteristics of all included individuals that reported exposure to vibrating hand-held tools as “not at all”, “some” and “much”.

	**“Does your work involve working with vibrating hand-held tools?”**			
	**“Not at all”**	**”Some”**	**”Much”**	**P-value**
	**12,220 (85%)**	**1,392 (10%)**	**730 (5%)**	
Age (years)	57±6	57±6	57±6	0.18
Sex (men)	4845 (40%)	1003 (72%)	601 (82%)	**<0.001**†,‡,§
Current smoking	3458 (28%)	477 (34%)	257 (35%)	**<0.001**†,‡
Antihypertensive treatment	5763 (47%)	708 (51%)	373 (51%)	**0.005**†,‡
Prevalent diabetes	546 (5%)	85 (6%)	37 (5%)	**0.02**†
Alcohol consumption (g/day)	7.3 [1.7-15.5]	8.1 [1.8-17.3]	9.0 [1.4-18.6]	**0.025**‡
Incident carpal tunnel syndrome (CTS)	460 (3.8%)	43 (3.1%)	29 (4.0%)	0.42
Incident ulnar nerve entrapment at elbow/wrist (UNE)	84 (0.7%)	16 (1.1%)	11 (1.5%)	**0.012**‡
Incident Dupuytren’s contracture	245 (2.0)	32 (2.3%)	18 (2.5%)	0.56

For peer review only

Incident trigger finger	399 (3.3%)	41 (2.9%)	34 (4.7%)	0.091
Incident osteoarthritis of the thumb CMC-1 joint	297 (2.4%)	33 (2.4%)	17 (2.3%)	0.98
Any incident hand disorder	1,235 (10.1%)	135 (9.7%)	87 (11.9%)	0.24

P-value for good comparisons between the subjects are based on Kruskal Wallis and Mann-Whitney U-test as a posthoc test and Chi2 test for ordinal variables. † Statistical significance was found between "not at all" and "some". ‡ Statistical significance was found between "not at all" and "much". § Statistical significance found between "some" and "much".

For peer review only

STROBE 2007 (v4) checklist of items to be included in reports of observational studies in epidemiology*
Checklist for cohort, case-control, and cross-sectional studies (combined)

Section/Topic	Item #	Recommendation	Reported on page #
**Title and abstract**	1	(*a*) Indicate the study's design with a commonly used term in the title or the abstract	1-2
		(*b*) Provide in the abstract an informative and balanced summary of what was done and what was found	2-3
**Introduction**			
Background/rationale	2	Explain the scientific background and rationale for the investigation being reported	4-5
Objectives	3	State specific objectives, including any pre-specified hypotheses	5
**Methods**			
Study design	4	Present key elements of study design early in the paper	5
Setting	5	Describe the setting, locations, and relevant dates, including periods of recruitment, exposure, follow-up, and data collection	5
Participants	6	(*a*) *Cohort study*—Give the eligibility criteria, and the sources and methods of selection of participants. Describe methods of follow-up<br>*Case-control study*—Give the eligibility criteria, and the sources and methods of case ascertainment and control selection. Give the rationale for the choice of cases and controls<br>*Cross-sectional study*—Give the eligibility criteria, and the sources and methods of selection of participants	5-6
		(*b*) *Cohort study*—For matched studies, give matching criteria and number of exposed and unexposed<br>*Case-control study*—For matched studies, give matching criteria and the number of controls per case	5-6
Variables	7	Clearly define all outcomes, exposures, predictors, potential confounders, and effect modifiers. Give diagnostic criteria, if applicable	5-6
Data sources/ measurement	8*	For each variable of interest, give sources of data and details of methods of assessment (measurement). Describe comparability of assessment methods if there is more than one group	5-6
Bias	9	Describe any efforts to address potential sources of bias	19
Study size	10	Explain how the study size was arrived at	5
Quantitative variables	11	Explain how quantitative variables were handled in the analyses. If applicable, describe which groupings were chosen and why	5-6
Statistical methods	12	(*a*) Describe all statistical methods, including those used to control for confounding	6
		(*b*) Describe any methods used to examine subgroups and interactions	6
		(*c*) Explain how missing data were addressed	6
		(*d*) *Cohort study*—If applicable, explain how loss to follow-up was addressed<br>*Case-control study*—If applicable, explain how matching of cases and controls was addressed	6

For peer review only

		Cross-sectional study—If applicable, describe analytical methods taking account of sampling strategy	
		(*e*) Describe any sensitivity analyses	-
**Results**			
Participants	13*	(a) Report numbers of individuals at each stage of study—eg numbers potentially eligible, examined for eligibility, confirmed eligible, included in the study, completing follow-up, and analysed	7-15
		(b) Give reasons for non-participation at each stage	-
		(c) Consider use of a flow diagram	included
Descriptive data	14*	(a) Give characteristics of study participants (eg demographic, clinical, social) and information on exposures and potential confounders	7-15
		(b) Indicate number of participants with missing data for each variable of interest	7-15
		(c) *Cohort study*—Summarise follow-up time (eg, average and total amount)	
Outcome data	15*	*Cohort study*—Report numbers of outcome events or summary measures over time	
		Case-control study—Report numbers in each exposure category, or summary measures of exposure	7-15
		Cross-sectional study—Report numbers of outcome events or summary measures	
Main results	16	(*a*) Give unadjusted estimates and, if applicable, confounder-adjusted estimates and their precision (eg, 95% confidence interval). Make clear which confounders were adjusted for and why they were included	7-15
		(*b*) Report category boundaries when continuous variables were categorized	7-15
		(*c*) If relevant, consider translating estimates of relative risk into absolute risk for a meaningful time period	
Other analyses	17	Report other analyses done—eg analyses of subgroups and interactions, and sensitivity analyses	-
**Discussion**			
Key results	18	Summarise key results with reference to study objectives	15-16
Limitations	19	Discuss limitations of the study, taking into account sources of potential bias or imprecision. Discuss both direction and magnitude of any potential bias	19
Interpretation	20	Give a cautious overall interpretation of results considering objectives, limitations, multiplicity of analyses, results from similar studies, and other relevant evidence	15-18
Generalisability	21	Discuss the generalisability (external validity) of the study results	19
**Other information**			
Funding	22	Give the source of funding and the role of the funders for the present study and, if applicable, for the original study on which the present article is based	20

*Give information separately for cases and controls in case-control studies and, if applicable, for exposed and unexposed groups in cohort and cross-sectional studies.

Note: An Explanation and Elaboration article discusses each checklist item and gives methodological background and published examples of transparent reporting. The STROBE checklist is best used in conjunction with this article (freely available on the Web sites of PLoS Medicine at http://www.plosmedicine.org/, Annals of Internal Medicine at http://www.annals.org/, and Epidemiology at http://www.epidem.com/). Information on the STROBE Initiative is available at www.strobe-statement.org.

For peer review only
